# Amino-acid selective isotope labeling enables simultaneous overlapping signal decomposition and information extraction from NMR spectra

**DOI:** 10.1007/s10858-019-00295-9

**Published:** 2020-01-30

**Authors:** Takuma Kasai, Shunsuke Ono, Seizo Koshiba, Masayuki Yamamoto, Toshiyuki Tanaka, Shiro Ikeda, Takanori Kigawa

**Affiliations:** 1 Laboratory for Cellular Structural Biology, RIKEN Center for Biosystems Dynamics Research, Yokohama, Japan; 2 PRESTO, JST, Kawaguchi, Japan; 3 School of Computing, Tokyo Institute of Technology, Yokohama, Japan; 4 Tohoku Medical Megabank Organization, Tohoku University, Sendai, Japan; 5 Graduate School of Medicine, Tohoku University, Sendai, Japan; 6 Department of Systems Science, Graduate School of Informatics, Kyoto University, Kyoto, Japan; 7 Department of Statistical Inference and Mathematics, The Institute of Statistical Mathematics, Tachikawa, Japan

**Keywords:** Combinatorial selective labeling, Stable isotope encoding (SiCode), Tensor factorization, Spectral deconvolution, Non-uniform sampling (NUS), Relaxation analysis

## Abstract

Signal overlapping is a major bottleneck for protein NMR analysis. We propose a new method, stable-isotope-assisted parameter extraction (SiPex), to resolve overlapping signals by a combination of amino-acid selective isotope labeling (AASIL) and tensor decomposition. The basic idea of Sipex is that overlapping signals can be decomposed with the help of intensity patterns derived from quantitative fractional AASIL, which also provides amino-acid information. In SiPex, spectra for protein characterization, such as ^15^N relaxation measurements, are assembled with those for amino-acid information to form a four-order tensor, where the intensity patterns from AASIL contribute to high decomposition performance even if the signals share similar chemical shift values or characterization profiles, such as relaxation curves. The loading vectors of each decomposed component, corresponding to an amide group, represent both the amino-acid and relaxation information. This information link provides an alternative protein analysis method that does not require “assignments” in a general sense; i.e., chemical shift determinations, since the amino-acid information for some of the residues allows unambiguous assignment according to the dual selective labeling. SiPex can also decompose signals in time-domain raw data without Fourier transform, even in non-uniformly sampled data without spectral reconstruction. These features of SiPex should expand biological NMR applications by overcoming their overlapping and assignment problems.

**Electronic supplementary material:**

The online version of this article (10.1007/s10858-019-00295-9) contains supplementary material, which is available to authorized users.

## Introduction

Signal overlapping is almost always an issue in NMR analyses of biomacromolecular structures and functions, because it hampers precise quantifications of signal positions, line widths, and/or intensities. For example, ^15^N relaxation measurements, the most widely used method for protein dynamics analysis, require accurate readouts of the signal intensities in the 2D ^1^H–^15^N correlation spectra to extract the relaxation rate constants (Kay et al. 1989; Clore et al. 1990). Therefore, the information for the residues of interest cannot be obtained unless their signal overlapping is resolved (Gümral et al. 2013).

This problem can be straightforwardly resolved by introducing additional dimensions. For example, a three-dimensional HNCO-type experiment can resolve the signal overlapping on the ^1^H–^15^N plane of ^15^N relaxation measurements if the carbonyl ^13^C chemical shifts are well separated (Caffrey et al. 1998). Its unfavorable longer measurement time can be fairly overcome by reducing the sampled data points, such as by non-uniform sampling (NUS) methods (Hyberts et al. 2014; Mobli and Hoch 2014; Kazimierczuk and Orekhov 2015). However, the applicable methods are limited because the signal intensity accuracy of the reconstructed NUS data is generally less reliable (Mayzel et al. 2017).

Another approach proposed by Orekhov et al. named MUNIN (Multi-dimensional NMR spectra interpretation), uses polyadic decomposition (PD), which can decompose tensors with three or more dimensions to the sum of the direct products of vectors. It was first applied to cross peaks in a crowded region of a 3D NOESY-HSQC spectrum (Orekhov et al. 2001), and then to the ^15^N R_1ρ_ relaxation measurements, by considering a series of 2D spectra as 3D data (Korzhnev et al. 2001). In the latter case, the overlapping signal on the ^1^H–^15^N dimensions generally exhibits a different relaxation curve along the third “relaxation” dimension, enabling the separation of the signals (Korzhnev et al. 2001).

Reducing the number of observable signals is a totally different type of approach to the signal overlapping problem. For this purpose, amino-acid selective isotope labeling (AASIL) is widely used for protein NMR (Vajpai et al. 2008), since it also facilitates the signal assignments by providing amino-acid type information (Yamazaki et al. 1991; Jaipuria et al. 2012). Especially, dual selective labeling (DSL) of both carbon and nitrogen greatly contributes to the main-chain signal assignments (Kainosho and Tsuji 1982). In general, more signal reduction results in less information obtained per labeled sample (Hoffmann et al. 2018). This trade-off relationship has led to a variety of DSL methods, from a single signal observation by labeling only one amino acid pair per sample (Yabuki et al. 1998) to more comprehensive observations of amide signals using a combination of differently labeled samples to represent a set of amino acids (combinatorial selective labeling; CSL) (Shortle 1994; Parker et al. 2004; Shi et al. 2004; Trbovic et al. 2005; Ozawa et al. 2006; Staunton et al. 2006; Wu et al. 2006; Reckel et al. 2008; Xun et al. 2009; Maslennikov et al. 2010; Sobhanifar et al. 2010; Hefke et al. 2011; Krishnarjuna et al. 2011; Jaipuria et al. 2012; Klammt et al. 2012; Löhr et al. 2012, 2014, 2015; Maslennikov and Choe 2013; Dubey et al. 2016; Laguerre et al. 2016; Hein et al. 2017; Hoffmann et al. 2018; Myshkin et al. 2019). We previously proposed one of the CSL methods, SiCode (Stable isotope encoding), which emphasizes the information content per sample to distinguish the 19 non-proline amino acids, with as few as three labeled samples, by quantitative fractional labeling (Kasai et al. 2015). In this method, the differences in the signal intensities among the samples not only provide the amino acid information but also contribute to the resolution of overlapping signals (Kasai et al. 2016).

In this paper, we propose a method for protein NMR employing AASIL and tensor decomposition of the spectrum, named SiPex (Stable-isotope-assisted Parameter extraction). Selective labeling, introduced as an extra dimension in SiPex, improves the decomposition of overlapped signals, as compared to methods using a uniformly labeled sample such as MUNIN (Korzhnev et al. 2001). Furthermore, SiPex provides an alternate strategy for protein NMR analysis that is independent of the chemical shifts, since it directly links the amino-acid and relaxation information of the decomposed signals.

## Theory

PD, also known as CP (canonical polyadic) decomposition, PARAFAC (parallel factor analysis), CANDECOMP (canonical decomposition), three-way decomposition (TWD), or multi-dimensional decomposition (MDD), is a method to decompose a tensor to a sum of outer products of vectors (Bro 1997; Orekhov et al. 2001). The PD of a three-order tensor is formulated as

1 $${\mathbf{\underline{X}}} = \sum\limits_{f = 1}^{F} {{\mathbf{a}}_{f} \otimes {\mathbf{b}}_{f} \otimes {\mathbf{c}}_{f} } + {\mathbf{\underline{E}}}$$

where ${\mathbf{\underline{X}}}$ is the observed data as a three-order tensor, *f* is an index of components, *F* is the number of components, **a**_*f*_, **b**_*f*_, and **c**_*f*_ are loading vectors (also called loads, shapes, or modes) (Bro 1997; Orekhov et al. 2001), $\otimes$ denotes the outer product, and ${\mathbf{\underline{E}}}$ is the residual error as a three-order tensor. PD is applied to tensors with three or more orders because the PD solution of a two-order tensor (i.e., matrix) is not unique due to so-called rotational ambiguity, which means that the components are mixed (Bro 1997; Orekhov et al. 2001). In contrast, the PD solution of tensors with three or more orders is unique, except for scaling and sign ambiguities (Bro 1997; Orekhov et al. 2001).

MUNIN (Korzhnev et al. 2001; Orekhov et al. 2001) utilizes PD to separate signals from NMR spectra. To measure the ^15^N R_1ρ_ relaxation rate constants, a set of 2D ^1^H–^15^N correlation spectra with different relaxation time delays and spin-lock offsets is regarded as a single three-order tensor (Korzhnev et al. 2001), so that the tensor can be uniquely decomposed by PD,

2 $${\mathbf{\underline{X}}}_{\text{MUNIN}}= \sum\limits_{f = 1}^{F} {{\mathbf{a}}_{f} \otimes {\mathbf{b}}_{f} \otimes {\mathbf{d}}_{f} } + {\mathbf{\underline{E}}}$$

where *f* is an index for an amide signal, **a**_*f*_ and **b**_*f*_ are the loading vectors along the ^1^H and ^15^N dimensions, respectively, and **d**_*f*_ is a loading vector representing the relaxation curve, which can be analyzed by standard exponential fitting. This method is also applicable to other experiments using amides as probes, such as measurements of relaxation properties including R_1_, R_2_, and heteronuclear ^1^H–^15^N NOE enhancements (Korzhnev et al. 2001). It should be noted that the relaxation curve **d**_*f*_ not only provides the relaxation properties of the amides but also serves as a clue for signal decomposition, when it differs between components.

The determination of amino acid type (amino acid typing) using SiCode is achieved by the comparison of the signal intensities of a set of 2D ^1^H–^15^N correlation spectra, ^15^N-HSQC and HN(CO), acquired using quantitatively labeled samples (Kasai et al. 2015). The intensity of the *f*-th amide signal of ^15^N-HSQC, ${\mathbf{c}}_{{{\text{HSQC}}\,f}} \in {\mathbb{R}}^{S}$, is proportional to the ^15^N labeling ratio:

3 $${\mathbf{c}}_{{{\text{HSQC}}\,f}} = w_{{{\text{HSQC}}\,f}} {\mathbf{r}}_{{\text{N}}} (p_{f} )$$

where *w*_HSQC*f*_ is the ^15^N HSQC intensity of the *f*-th amide signal, assuming that the amide is uniformly ^15^N labeled, ${\mathbf{r}}_{{\text{N}}} (p) \in \left[ {0,1} \right]^{S}$ is a vector representing the ^15^N labeling ratios of amino acid *p*, *p*_*f*_ is the amino acid type of the *f*-th amide signal (“residue i”), and *S* is the number of labeled samples. The intensity of the *f*-th amide signal of HN(CO), ${\mathbf{c}}_{{{\text{HNCO}}\,f}} \in {\mathbb{R}}^{S}$, is proportional to the product of the ^15^N labeling ratio and the ^13^C labeling ratio:

4 $${\mathbf{c}}_{{{\text{HNCO}}\,f}} = w_{{{\text{HNCO}}\,f}} {\mathbf{r}}_{{\text{N}}} (p_{f} ) \circ {\mathbf{r}}_{{\text{C}}} (q_{f} )$$

where *w*_HNCO*f*_ is the HN(CO) intensity of the *f*-th amide signal when the amide is 100% ^15^N labeled and the carbonyl of the preceding residue (“residue i-1”) is 100% ^13^C labeled, ${\mathbf{r}}_{{\text{C}}} (q) \in \left[ {0,1} \right]^{S}$ is a vector representing the ^13^C labeling ratios of amino acid *q*, *q*_*f*_ is the amino acid type of the residue i-1 of the *f*-th amide signal, and $\circ$ denotes the element-wise product. Amino acid typing by SiCode shares the similar structure of the problem with the determination of relaxation properties; i.e., the signal intensities among 2D spectra contain information. Therefore, signal decomposition and amino acid typing using SiCode are also achieved using PD in a similar manner to Eq. 2, by regarding a set of 2D spectra as a single three-order tensor,

5 $${\mathbf{\underline{X}}}_{\text{SiCode}} = \sum\limits_{f = 1}^{F} {{\mathbf{a}}_{f} \otimes {\mathbf{b}}_{f} \otimes {\mathbf{c}}_{f} } + {\mathbf{\underline{E}}}$$

6 $${\mathbf{c}}_{f} = \left( {\begin{array}{*{20}c} {{\mathbf{c}}_{{{\text{HSQC}}\,f}} } \\ {{\mathbf{c}}_{{{\text{HNCO}}\,f}} } \\ \end{array} } \right)$$

where *f* is an index for an amide signal, **a**_*f*_ and **b**_*f*_ are the loading vectors along the ^1^H and ^15^N dimensions, respectively, and ${\mathbf{c}}_{f} \in {\mathbb{R}}^{2S}$ is a loading vector representing the intensity difference between spectra. The estimations of the amino-acid types of the *f*-th amide signals, $\hat{p}_{f}$ at residue i and $\hat{q}_{f}$ at residue i-1, can be obtained from **c**_*f*_ by minimizing the residual error:

7 $$(\hat{p}_{f} ,\hat{w}_{{{\text{HSQC}}\,f}} ) = \mathop {\arg \min }\limits_{{p_{f} ,w_{{{\text{HSQC}}\,f}} }} \left\| {{\mathbf{c}}_{{{\text{HSQC}}\,f}} - w_{{{\text{HSQC}}\,f}} {\mathbf{r}}_{{\text{N}}} (p_{f} )} \right\|_{2}^{2}$$

8 $$(\hat{q}_{f} ,\hat{w}_{{{\text{HNCO}}\,f}} ) = \mathop {\arg \min }\limits_{{q_{f} ,w_{{{\text{HNCO}}\,f}} }} \left\| {{\mathbf{c}}_{{{\text{HNCO}}\,f}} - w_{{{\text{HNCO}}\,f}} {\mathbf{r}}_{{\text{N}}} (\hat{p}_{f} ) \circ {\mathbf{r}}_{{\text{C}}} (q_{f} )} \right\|_{2}^{2}$$

Based on these operations, we can convert the intensities of the loading vector along the SiCode dimension into the amino acid information by selecting the amino acid with the nearest labeling ratios to the intensities.

SiPex integrates the amino acid typing and the relaxation measurement by acquiring relaxation spectra with SiCode samples to form a PD problem of a four-order tensor:

9 $${\mathbf{\underline{\underline{X}}}} = \sum\limits_{f = 1}^{F} {{\mathbf{a}}_{f} \otimes {\mathbf{b}}_{f} \otimes {\mathbf{c}}_{f} \otimes {\mathbf{d}}_{f} } + {\mathbf{\underline{\underline{E}}}}$$

where ${\mathbf{\underline{\underline{X}}}}$ and ${\mathbf{\underline{\underline{E}}}}$ are four-order tensors.

This integration gains two advantages. The first is that increasing the number of dimensions simply reduces the risk of decomposition failure. In SiPex, if overlapping signals share the identical (or virtually identical) loading vectors in at most one of the four dimensions, then the other three dimensions are mathematically sufficient to avoid the rotational ambiguity (Orekhov et al. 2001).

The second is that it also provides an alternative to conventional protein analysis, where the signals in the relaxation spectra must be identified using previously determined chemical shift values. In contrast, each decomposed component of SiPex contains both amino-acid information, **c**_*f*_, and relaxation information, **d**_*f*_. According to the DSL approach (Kainosho and Tsuji 1982; Yabuki et al. 1998), the residues of an amino acid pair (residues i and i-1) that occurs only once in the sequence can be unambiguously assigned with the amino acid information. Therefore, the relaxation properties are obtained along with the assignments for such residues, even without the chemical shift information.

## Results and discussion

### Applicability of tensor decomposition to the SiCode dataset to determine amino-acid types

**Fig. 1:**
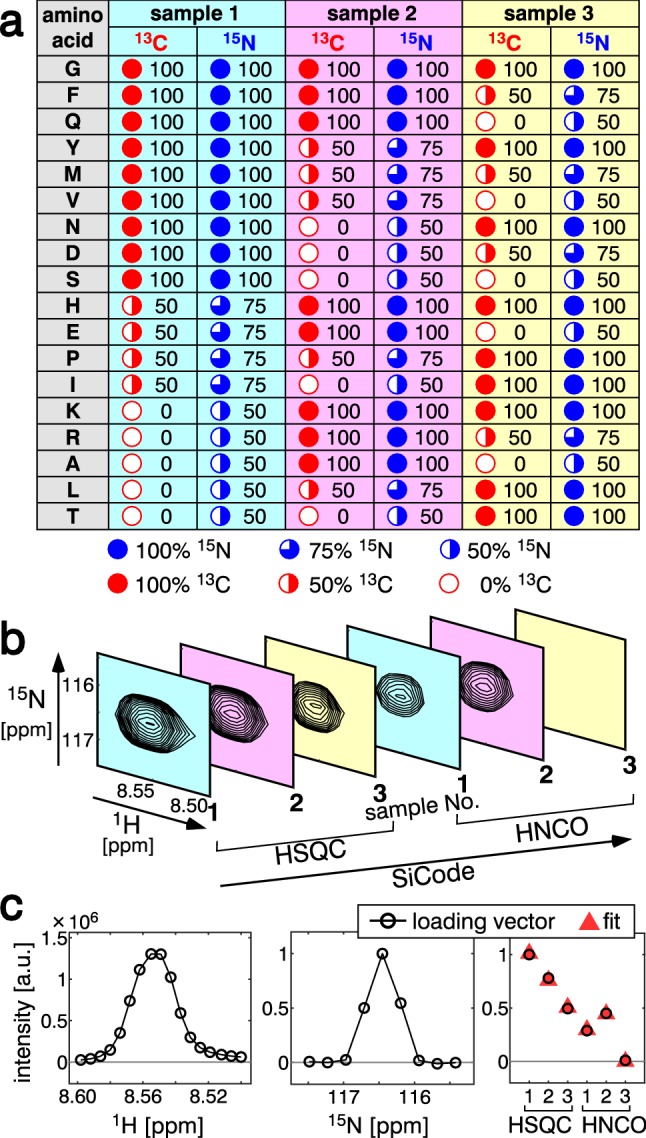
Encoding and decoding amino acid information for amide signals. **a** The labeling pattern (“codebook”) used in this study. Each amino acid is represented as a combination (a “codeword”) of the isotope labeling ratios of three labeled samples. The labeling ratios of ^13^C and ^15^N are indicated as percentages. **b** A set of 2D spectra to form a three-order tensor. A small region-of-interest (ROI) that contains the (E)V17 signal was extracted. **c** Loading vectors of the signal. From left to right, the loading vectors along the ^1^H, ^15^N, and SiCode dimensions are shown as black lines and circles. The best fits to the extracted amino-acid information (Eqs. 7and8) are shown as red triangles

At first, we demonstrate that the NMR spectra of SiCode samples are suitable for the decomposition to provide amino acid information, according to Eqs. 5–8. We prepared three samples of a human ubiquitin 3A mutant (Ub3A) protein according to a SiCode labeling pattern (Fig. 1a), which was designed to allow 18 amino acids (excluding cysteine and tryptophan, which did not appear in the Ub3A sequence) to be distinguished by the signal intensity differences. For the demonstration, a small region of interest (ROI) in the 2D ^1^H-^15^N plane containing the (E)V17 signal (amide signal derived from valine 17 preceded by glutamate 16) was extracted (Fig. 1b). The HSQC and HN(CO) spectra of the three samples were assembled as a single three-order tensor (Fig. 1b) with $17 \times 9 \times 6$ points along the ^1^H, ^15^N, and SiCode dimensions, respectively. We performed the PD of the tensor (Fig. 1c), using the alternating least squares (ALS) algorithm. The loading vector along the SiCode dimension, **c**_*f*_, contained the amino-acid information. We decoded the amino-acid information from **c**_*f*_ according to Eqs. 7–8. The first half of the vector, **c**_HSQC*f*_, indicated that the amino-acid at residue i was valine according to Eq. 7, to which we assigned ^15^N labeling ratios of 100%, 75%, and 50% (for samples 1–3, respectively, Fig. 1a), and the latter half, **c**_HNCO*f*_, indicated that the amino acid at residue i-1 was glutamate according to Eq. 8, to which we assigned ^13^C labeling ratios of 50%, 100%, and 0% (Fig. 1a). Note that the HN(CO) intensity of each sample was the product of the ^15^N labeling ratio at residue i and the ^13^C labeling ratio at residue i-1. We successfully extracted the correct amino-acid information for both residues i and i-1, suggesting that SiCode is a suitable labeling method for use in SiPex.

### Decomposition of overlapping signals and relaxation measurements

**Fig. 2:**
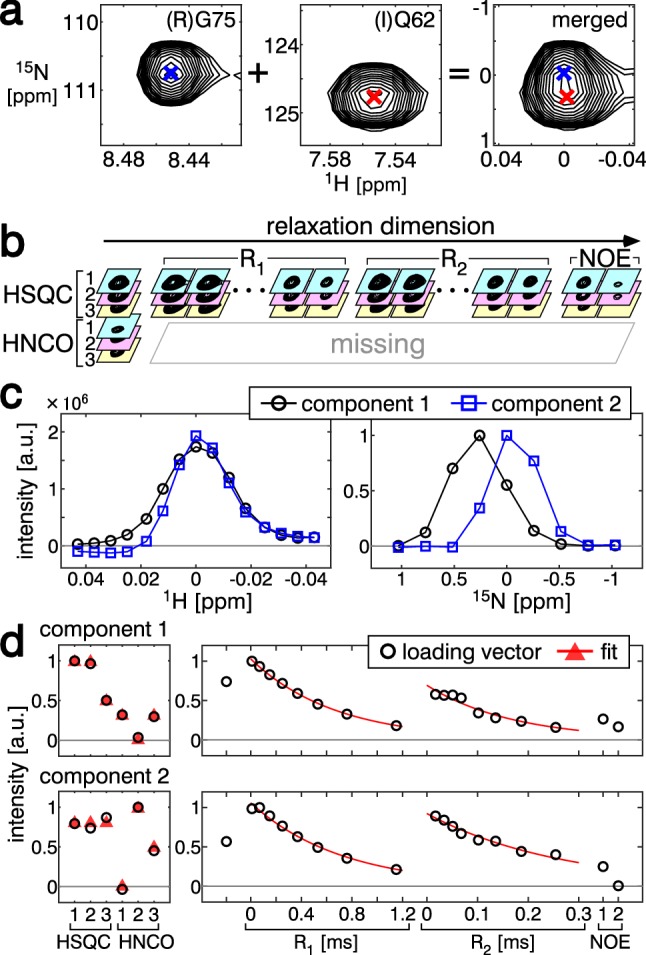
Decomposition of simulated overlapping signals. **a** Preparation of the artificial dataset with overlapping signals. ROIs with the same size, including the (R)G75 (indicated by blue crosses) and (I)Q62 (indicated by red crosses) signals, were extracted and merged by element-wise tensor addition. Only the ^15^N HSQC spectrum of sample 1 is shown. **b** Illustration of four-order tensor formation with a set of 2D spectra. **c** Loading vectors along the ^1^H (left) and ^15^N (right) dimensions. The first (*f* = 1) component is shown as black lines and circles, and the second (*f* = 2) component is shown as blue lines and squares. **d** Loading vectors along the SiCode (left) and relaxation (right) dimensions. The first (top panels) and second (bottom panels) components are shown. Red triangles and lines indicate the best fits for the extraction of amino acid information and the exponential decays

To test the capability of SiPex to decompose overlapping signals in a 2D ^1^H–^15^N plane, we simulated overlap in one merged ROI by the element-wise addition of two distinct ROIs, one including the (I)Q62 signal and the other including the (R)G75 signal of the Ub3A protein (Fig. 2a). The chemical shift differences of the two signals were 0.002 ppm and 0.35 ppm along the ^1^H and ^15^N dimensions, respectively. Due to this close overlap, signal decomposition is required to obtain the signal intensities or volumes for the relaxation analysis.

The loading vector **d**_*f*_ can accept any line shape, and thus several relaxation experiments can be analyzed simultaneously. In this study, we analyzed R_1_, R_2_, and the heteronuclear NOE measurements by setting **d**_*f*_ in Eq. 9 as

10 $${\mathbf{d}}_{f} = \left( {\begin{array}{*{20}c} {d_{{{\text{HSQC}}\,f}} } \\ {{\mathbf{d}}_{{{\text{R1}}\,f}} } \\ {{\mathbf{d}}_{{{\text{R2}}\,f}} } \\ {{\mathbf{d}}_{{{\text{NOE}}\,f}} } \\ \end{array} } \right)$$

where* d*_HSQC*f*_ is a scalar value not used in further analysis, **d**_R1*f*_ and **d**_R2*f*_ are the decay curves for the R_1_ and R_2_ measurements, respectively, and ${\mathbf{d}}_{{{\text{NOE}}\,f}} \in {\mathbb{R}}^{2}$ is a vector that corresponds to the intensities without and with irradiation for the steady-state NOE measurement. The four-order tensor used in this case is illustrated in Fig. 2b. In this case, we measured 8 different relaxation time delays for both the R_1_ and R_2_ measurements; therefore, the relaxation dimension comprises 19 (1 + 8 + 8 + 2) data points. In this study, ^15^N-HSQC and HNCO were acquired with a recycle delay of 1 s, while the relaxation spectra were acquired with a delay of 3 s to facilitate the magnetization recovery.

We decomposed the tensor by ALS, assuming the number of components *F* = 2 (Fig. 2c). After decomposition, the amino acid information can be decoded from **c**_*f*_. The decoded amino acids were (I)Q for component 1 (*f* = 1) and (R)G for component 2 (*f* = 2) (Fig. 2d). The amino acid information allowed us to identify components that were in an arbitrary order, depending on the random initial values of the loading vectors. We confirmed that the relaxation properties extracted from **d**_*f*_ (Fig. 2d) showed good agreement with those generated by the conventional method, which we separately obtained from isolated signals with a uniformly labeled sample (Table 1, “Simulated overlapping”).

**Table 1:** Amino acids and relaxation parameters of Ub3A obtained from overlapping signals by SiPex, compared with those obtained by the conventional method with the uniformly labeled sample

	Residue	SiPex					Conventional		
		i	i-1	R_1_ [s^−1^]	R_2_ [s^−1^]	NOE	R_1_ [s^−1^]	R_2_ [s^−1^]	NOE
Simulated overlapping	(I)Q62	Q	I	1.50	5.83	0.63	1.50	6.46	0.67
	(R)G75	G	R	1.39	3.75	0.02	1.45	3.77	0.13
Actual overlapping	(L)E16	E	L	1.59	7.08	0.74			
	(N)V26	N	V	1.75	6.87	0.80			

The actual number of components, *F*, is unknown at the time of decomposition. We tried decompositions with different *F* values. When *F* = 1 was assumed, some residual error remained (Note S1), suggesting that *F* was too small to explain the data. On the other hand, when *F* = 3 was assumed, the negative values of the loading vectors indicated overfitting (Note S1). These results suggested that *F* = 2 was the best assumption.

Intensity differences may hinder the decomposition. To simulate such situations, we multiplied the ROI with (R)G75 by 0.3 prior to the element-wise addition of ROIs. As a result, the (I)Q62 signal had a 2.8-fold larger intensity than (R)G75 in the ^15^N-HSQC spectrum. SiPex still successfully decomposed these signals to provide the amino-acid and relaxation information (Fig. S6).

We then applied the method to real overlapping signals, (L)E16 and (N)V26 (Fig. S7a). The signals were successfully decomposed by assuming *F* = 2 (Fig. S7b) and both the amino-acid and relaxation information were obtained (Table 1, “Actual overlapping”). We confirmed that *F* = 2 was a reasonable assumption by trials with different *F* values (Note S1). To evaluate the advantage of using SiCode samples, we performed a three-order tensor decomposition of the same ROI of spectra acquired with a uniformly-labeled sample. The three-order tensor decomposition, with only the ^1^H, ^15^N, and relaxation dimensions, failed to resolve these signals (Note S2). These results demonstrated that SiPex, fortified by selective labeling, can resolve the overlapping signals for the accurate estimation of the intensities of each signal, which are necessary for the relaxation measurements, even without time-consuming 3D experiments.

### Direct analysis of non-uniformly sampled time-domain data

In modern NMR, simultaneously excited magnetizations with different resonant frequencies are acquired as a mixture in time-domain raw data, and subsequently separated by a Fourier transform. The Fourier transform is performed for two purposes: one is to visually separate signals mixed in raw time-domain data, and another is to extract the resonance frequency; i.e., the chemical shift values, of each magnetization by converting the time domain to the frequency domain. For the former purpose, the tensor decomposition can also separate mixed signals in the time-domain data. For this reason, the methods using tensor decomposition, including SiPex and MUNIN (Orekhov et al. 2001), can directly handle the time-domain data without Fourier transform.

**Fig. 3:**
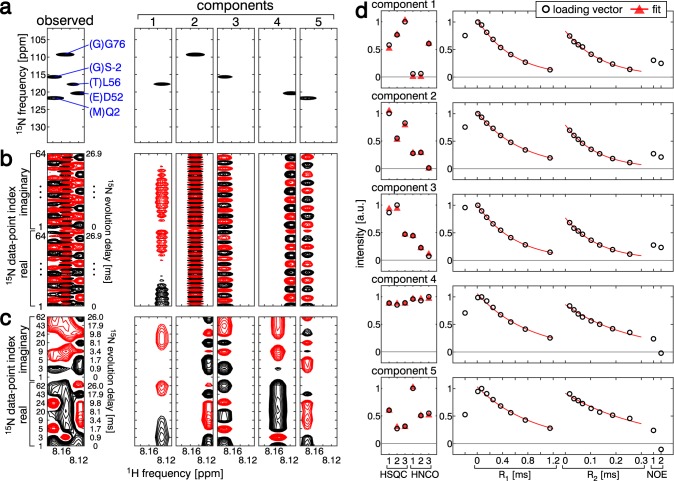
Analysis of non-uniformly sampled time domain data. **a** Decomposition of the ROI in which the ^15^N dimension is the Fourier transformed frequency domain. Black and red lines show positive and negative contours, respectively. The leftmost panel is the observed data and the right five panels are the decomposed components. Only the ^15^N HSQC spectrum of sample 1 is shown. **b** Decomposition of the same ROI as in (**a**), but the ^15^N dimension is the time-domain raw data. Both the real and imaginary parts of the complex data are shown. **c** A simulation of NUS in the ^15^N dimension, by extracting 8 out of the 64 complex points used in (**b**). **d** Loading vectors along the SiCode (left) and relaxation (right) dimensions. From top to bottom, five decomposed components are shown. The markers and line styles are the same as in Fig. 2d

We selected an ROI, in which the ^1^H chemical shift is 8.107–8.193 ppm, containing five signals, (G)S-2, (M)Q2, (E)D52, (T)L56, and (G)G76 (Fig. 3a). Before applying SiPex to the time-domain data, we tested it with the frequency-domain spectra. Assuming *F* = 5, we successfully decomposed the signals (Fig. 3a) and extracted the amino acid information and relaxation properties (Fig. S3f, Table 2 “SiPex Frequency domain”). For the frequency domain data, the good signal dispersion allowed us to extract the relaxation properties with the conventional method (Table 2 “Conventional Frequency domain”). In contrast, in the time-domain data before Fourier transform along the ^15^N dimension, the signals in the same ROI must be decomposed for further analyses (Fig. 3b). We applied SiPex to these time domain data, in which the real and imaginary parts of 64 complex data points were placed along the ^15^N dimension to form 128 real data points. SiPex successfully decomposed the signals, as in the case of the frequency domain (Figs. 3b, S4f, Table 2 “SiPex Time domain Full sampling”), demonstrating that SiPex accepts any line shape, including both the frequency- and time-domain, of the loading vector along the ^15^N dimension.

**Table 2:** Amino acids and relaxation parameters of Ub3A obtained with frequency- or time-domain spectra

	Residue	i	i-1	R_1_ [s^−1^]	R_2_ [s^−1^]	NOE
Conventional Frequency domain	(G)S-2			1.21	3.34	-0.30
	(M)Q2			1.68	7.22	0.79
	(E)D52			1.49	7.90	0.79
	(T)L56			1.87	7.80	0.82
	(G)G76			1.23	3.33	-0.15
SiPex Frequency domain	(G)S-2	S	G	1.09	3.04	-0.53
	(M)Q2	Q	M	1.67	7.50	0.85
	(E)D52	D	E	1.42	7.97	0.79
	(T)L56	L	T	1.75	7.67	0.80
	(G)G76	G	G	1.20	3.74	-0.13
SiPex Time domain Full sampling	(G)S-2	S	G	1.09	3.07	-0.51
	(M)Q2	Q	M	1.67	7.42	0.84
	(E)D52	D	E	1.42	7.92	0.79
	(T)L56	L	T	1.75	7.58	0.80
	(G)G76	G	G	1.20	3.71	-0.13
SiPex Time domain NUS	(G)S-2	S	G	1.11	3.09	-0.44
	(M)Q2	Q	M	1.69	7.41	0.85
	(E)D52	D	E	1.43	7.90	0.78
	(T)L56	L	T	1.76	6.93	0.82
	(G)G76	G	G	1.21	3.84	-0.09

NUS is widely used to shorten the measurement time by reducing the observed time-domain data points in the indirect dimension(s). We simulated the NUS dataset by extracting 8 complex points, in which the evolution delays for the ^15^N dimension were 0.0, 0.9, 1.7, 3.4, 8.1, 9.8, 17.9, and 26.0 ms (Fig. 3c), out of 64 observed points according to the exponentially weighted random sampling (Barna et al. 1987). The required number of data points depends on the number of components, the divergence of vectors along the other dimensions, and the distribution of signals along the ^15^N dimension. In general, fewer NUS data points increase the possibility of the degeneracy of the vectors along the ^15^N dimension, which makes the decomposition problem difficult. This subset of time-domain data was sufficient for the decomposition and extraction of information by SiPex (Fig. 3c, d, Table 2 “SiPex Time domain NUS”). We tried various numbers for the component *F*, and found that *F* = 5 was the best assumption (Note S1). The decomposition of the same ROI with the uniformly labeled sample failed to resolve the signals for both the frequency-domain and time-domain data, indicating that the SiCode dimension is crucial for the successful signal decomposition (Note S2).

In this study, the ^15^N indirect dimension alone was employed for the time-domain analysis. For the ^1^H direct dimension, data manipulation in the frequency domain is better than that in the time domain, for three reasons. Firstly, the direct dimension can be uniformly and fully sampled without wasting time. Secondly, the water signal is much easier to separate from the amide signals with Fourier transform. Thirdly, the ROI is easier to extract, to reduce the computation time.

Each component decomposed by SiPex contains the amino acid information of residues i and i-1, as well as the relaxation properties of residue i. The amino acid information is sufficient to unambiguously assign part of the amide signals, according to the DSL approach (Kainosho and Tsuji 1982; Yabuki et al. 1998). Therefore, for such residues, (M)Q2, (E)D52, and (G)G76 in this case, we can obtain the relaxation properties without determining the chemical shift values. This provides an alternative strategy to protein relaxation analysis by skipping the chemical shift determination, which is essential to interpret the relaxation spectra in the conventional method. It should be noted that this strategy still assigns signals, and is therefore distinct from methods that extract information by statistical analysis without signal assignments (Rumpel et al. 2008; Kodama et al. 2013; Arbogast et al. 2017).

In general, NUS is used in combination with spectral reconstruction methods (Barna and Laue 1987; Daniell and Hore 1989; Hoch et al. 1990; Tugarinov et al. 2005; Hyberts et al. 2007; Stern et al. 2007; Coggins and Zhou 2008; Matsuki et al. 2009; Holland et al. 2011; Kazimierczuk and Orekhov 2011; Bostock et al. 2012; Hyberts et al. 2012; Sun et al. 2015; Ying et al. 2017), in which signal properties such as intensities, chemical shifts, and line widths are not guaranteed to be properly retained. The direct analysis of NUS data is not affected by the reconstruction quality, and therefore is a more straightforward method. The disadvantage of the direct NUS data analysis without the chemical shift information is partly compensated in SiPex, because of its ability to assign signals without chemical shift values based on DSL.

### Analysis of an intrinsically disordered protein

**Fig. 4:**
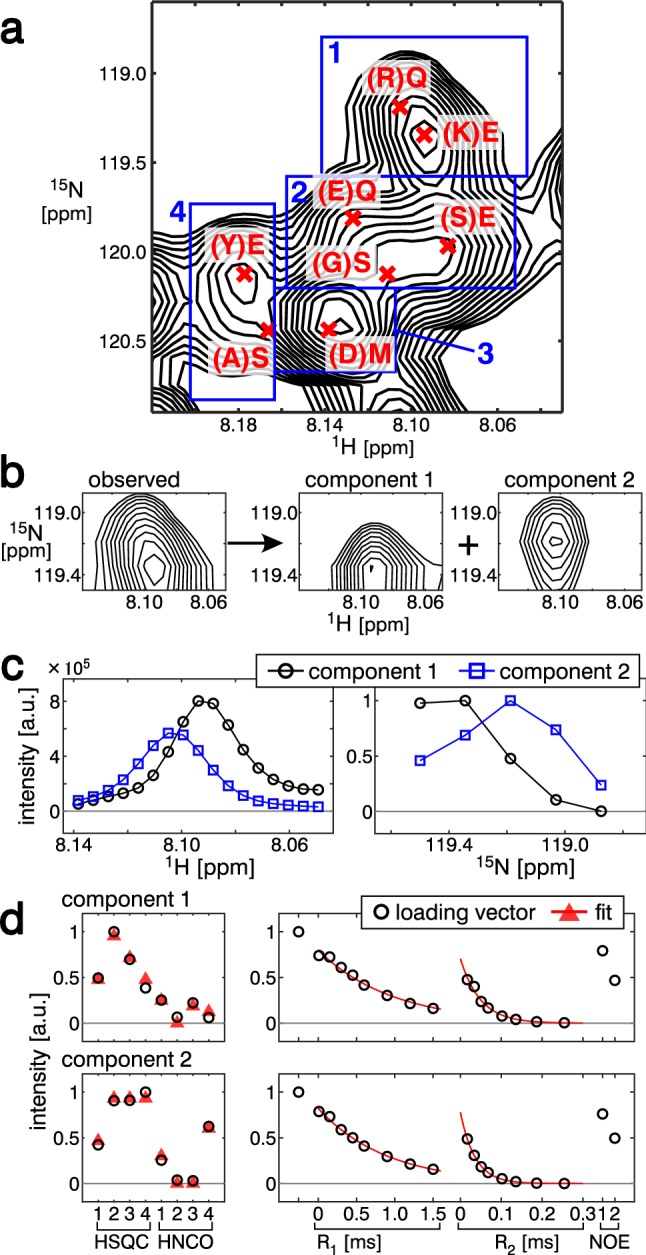
Analysis of a crowded spectral region of an intrinsically disordered protein. **a**^15^N HSQC spectrum of sample 1 of the analyzed spectral region. Four subregions for individual tensor decomposition runs are shown in the blue box and numbered. The decomposed signals are shown by red crosses with amino-acid information by SiPex. **b**–**d** Decomposition of subregion 1. **b** The observed spectrum (left panel) was decomposed into two components (right two panels). Only the ^15^N HSQC spectrum of sample 1 is shown. **c** Loading vectors along the ^1^H (left) and ^15^N (right) dimensions. The markers and line styles are the same as in Fig. 2c. **b** Loading vectors along the SiCode (left) and relaxation (right) dimensions. The markers and line styles are the same as in Fig. 2d

Intrinsically disordered proteins (IDPs) show limited dispersion of NMR signals (Alderson and Markley 2013), which are difficult to separate even by introducing an additional dimension, such as 3D HNCO-based ^15^N relaxation experiments. Since SiPex provides a different signal separation strategy, it would potentially be suitable for IDP analyses. As a model IDP, we analyzed the 98-amino-acid Nrf2-ECH-homology 2 (Neh2) domain of the nuclear factor E2-related factor 2 (Nrf2) protein (Itoh et al. 1999). We prepared four samples according to the labeling pattern shown in Fig. S12. The crowded region shown in Fig. 4a was divided into 4 subregions. By tensor decompositions of each subregion, we obtained 8 signals accompanied with amino-acid and relaxation information (Figs. 4, S13). The decomposition of 2 signals in subregion 1 is demonstrated in Fig. 4b–d. In subregion 1, we found two components with the amino acid types (K)E and (R)Q, confirmed as (K)E57 and (R)Q59, respectively, by separately performed sequential assignments using 3D triple-resonance experiments. The loading vectors and the amino-acid and relaxation information of all of the subregions are shown in Fig. S13. In total, 5 out of the 8 signals confirmed that the amino acids were correct ((D)M17, (Y)E47, (K)E57, (R)Q59, and (E)Q66); 2 signals ((A)S and (G)S) could not be confirmed due to the sensitivity issue of the triple-resonance experiments; and the other signal ((S)E) was incorrect because two overlapping signals ((L)E55, (K)E65) were wrongly treated as a single signal. The assigned 4 signals, (Y)E47, (K)E57, (R)Q59, and (E)Q66, showed similar relaxation parameters to each other (Table 3). The relaxation parameters of the other assigned signal, (D)M17, were similar to those of an isolated signal, (D)L19, which was a closely located residue in the primary sequence (Table 3). These results imply the reliability of the relaxation parameters obtained from the overlapping signals. In this study, the labeling pattern was only used to convert the intensities of the loading vector into the amino-acid information after the decomposition. A tensor decomposition algorithm using prior knowledge of the labeling pattern should improve the decomposition performance for highly crowded spectral regions, in the cases of IDPs. Although further improvement is required, the results showed that SiPex can potentially be applied to IDPs.

**Table 3:** Amino acids and relaxation parameters of Nrf2 Neh2 obtained by SiPex

	Residue	i	i-1	R_1_ [s^−1^]	R_2_ [s^−1^]	NOE
Overlapping	(D)M17	M	D	1.28	9.2	0.44
	(Y)E47	E	Y	1.10	22.5	0.66
	(K)E57	E	K	1.03	20.4	0.59
	(R)Q59	Q	R	1.12	27.7	0.65
	(E)Q66	Q	E	1.10	20.3	0.51
Isolated	(D)L19	L	D	1.40	8.2	0.45

### Application and further improvement of SiPex

SiPex decomposes overlapping signals depending on the difference in the loading vectors, which also contain information of interest. In this study, we employed SiCode as a selective labeling method because the amino acid information is represented as the signal intensities (Fig. 1b), and thus it can be straightforwardly regarded as the loading vector. Other CSL methods, in which the amino acid information is deduced from the visual inspection of the presence or absence of signals (Trbovic et al. 2005; Maslennikov et al. 2010; Jaipuria et al. 2012; Dubey et al. 2016; Hoffmann et al. 2018), can also be analyzed in the same way as SiCode, by regarding them as a special case of SiCode using only 0% or 100% labeling. Therefore, the analysis method proposed in this study can be used with other CSLs, as well as SiCode. We employed SiCode as the most suitable labeling method for SiPex, because it requires a smaller number of samples than other CSLs to distinguish all of the amino acids (Kasai et al. 2015). In other words, if the number of samples is limited, for example 3, some amino acids must share the same labeling pattern in other CSLs, which leads to a loss of amino acid information and reduced decomposition performance.

In this study, we integrated the relaxation measurements into SiPex by regarding the intensities of multiple spectra with different relaxation delays as a loading vector. SiPex is also applicable to other experiments using signal intensity modulation in a similar manner, such as paramagnetic relaxation enhancement (Otting 2010; Clore 2015), hydrogen–deuterium exchange (Englander and Mayne 1992), and cross saturation (Shimada 2005).

Further improvement of the decomposition algorithm will be required for more difficult targets. In this study, we used a simple, in-house ALS program (see Materials and Methods). For practical use, we recommend using software packages for tensor decomposition that run more efficiently by various acceleration methods; for example, the N-way toolbox (Andersson and Bro 2000), with which we obtained the same results as those presented in this paper (Note S3). By using such general-purpose tensor decomposition software, it may be possible to improve the decomposition in difficult cases by applying appropriate regularization based on the background knowledge; for example, non-negativity of the signals in the frequency domain and the labeling ratios. To further improve the decomposition performance, a specialized program using background knowledge more specific for SiPex, such as the labeling pattern or the exponential decay property in relaxation measurements, is promising. Optimizations of the experimental settings, such as the allocation balance on machine time among experiments (HSQC, HNCO, and relaxation) and the sampling schedule for NUS, also remain as subjects for future investigation.

## Conclusion

SiPex integrates two types of separately performed experiments, amino acid typing with AASIL and characterization of the target protein, by tensor decomposition of the whole data. This not only improved the separation of overlapping signals but also directly linked the information of the amino acid and the characteristics without chemical shift values. When combined with signal assignments based on the DSL approach, SiPex provides an alternative method for protein analysis without chemical shift determination. The basic concept presented here will potentially expand NMR applications by solving the problems in signal assignments and overlapping decomposition for difficult target proteins.

## Materials and methods

### Sample preparation

The amino acid sequence of Ub3A is the same as residues 1 to 76 of UniProt P62979, except for three alanine substitutions (L8A, I44A, and V70A) and an additional aspartate residue at its C-terminus (Inomata et al. 2009). The amino acid sequence of Nrf2 Neh2 is the same as residues 1 to 98 of UniProt Q60795. The template DNA encoding Ub3A or Neh2, with a histidine affinity tag sequence at its N-terminus, was constructed as previously described (Yabuki et al. 2007). Three (Ub3A) or four (Neh2) proteins, selectively labeled according to the SiCode labeling pattern shown in Fig. 1a (Ub3A) or Fig. S12 (Neh2), were produced with the dialysis mode of the cell-free protein synthesis system (Kigawa et al. 1995, 2004; Matsuda et al. 2007; Kigawa 2010), with the inhibition of amino acid scrambling (Yokoyama et al. 2011) as described previously (Kasai et al. 2015), using the amino acid mixture described in Table S1 (Ub3A) or Table S2 (Neh2). The proteins were purified with a HisTrap affinity column (GE Healthcare) and subsequently cleaved with tobacco etch virus protease. The cleaved Ub3A protein contains seven additional residues (GSSGSSG) as a cloning artifact at its N-terminus, which are represented by negative residue numbers in this paper. The cleaved Neh2 protein contains an additional glycine residue as a cloning artifact at its N-terminus. The proteins were finally purified with a HiTrap SP cation exchange column (GE Healthcare) (Ub3A) or a HiTrap Q anion exchange column (GE Healthcare) (Neh2). The purified proteins were concentrated to 1 mM in measurement buffer (50 mM sodium phosphate, pH 6.0, 10% ^2^H_2_O for Ub3A, 20 mM sodium phosphate, pH 8.0, 100 mM sodium chloride, 10% ^2^H_2_O for Neh2) by ultrafiltration with VIVASPIN 2 MWCO 5,000 concentrators (Sartorius).

### NMR measurements

All NMR measurements were performed with an AVANCE III HD 700 MHz spectrometer (Bruker BioSpin) equipped with a TCI (Ub3A) or a TXI (Neh2) CryoProbe (Bruker BioSpin) at 298 K at a 1 mM protein concentration. Two-dimensional ^15^N-HSQC, HNCO, ^15^N-R_1_-HSQC, ^15^N-R_2_-HSQC, and ^1^H-^15^N heteronuclear NOE-HSQC spectra were acquired. All NMR data were processed with the NMRPipe software (Delaglio et al. 1995). The number of data points, the spectral widths, and the carrier frequencies were the same for all of the measurements (Tables S3, S4). The delay times for the ^15^N R_1_ measurements were 10, 70, 150, 250, 370, 530, 760, and 1,150 ms (Ub3A) or 10, 150, 300, 450, 600, 900, 1,200, and 1,500 ms (Neh2). The durations of the CPMG sequence for the ^15^N R_2_ measurements were 17.0, 33.9, 50.9, 67.8, 101.8, 135.7, 186.6, and 254.4 ms. Before tensor decomposition, the spectra were multiplied by constant values to compensate for the concentration differences among the samples (See Note S4 for details).

### Tensor decomposition with alternating least squares (ALS)

In the sub-problem of ALS, the loading vector along one dimension is solved while assuming that the loading vectors along the other dimensions are known. Each element of the four-order tensor ${\mathbf{\underline{\underline{X}}}}$ in Eq. 9 can be written as:

11 $$x_{ijkl} = \sum\limits_{f = 1}^{F} {a_{if} b_{jf} c_{kf} d_{lf} } + e_{ijkl} \quad (i = 1 \ldots I,j = 1 \ldots J,k = 1 \ldots K,l = 1 \ldots L)$$

where *I*, *J*, *K*, and *L* are the number of data points along each dimension. To solve **a**_*f*_ as a sub-problem, the other three dimensions are unfolded to one dimension by introducing the new index, *m* = 1…*M*, *M* = *JKL*,

12 $$x_{im} = \sum\limits_{f = 1}^{F} {a_{if} z_{fm} } + e_{im} \quad x_{im} = x_{ijkl} ,z_{fm} = b_{jf} c_{kf} d_{lf} ,{\kern 1pt} e_{im} = e_{ijkl}$$

or in matrix representation,

13 $${\mathbf{X}} = {\mathbf{AZ}} + {\mathbf{E}}$$

where **X** and **E** are $I \times M$ matrices, **A** is an $I \times F$ matrix, and **Z** is an $F \times M$ matrix. The sub-problem is to find **A** that minimizes the sum of squares of elements of **E**, when **X** and **Z** are known. **A** is updated as

14 $${\mathbf{A}} = {\mathbf{XZ}}^{{\text{T}}} ({\mathbf{ZZ}}^{{\text{T}}} )^{ - 1}$$

In one iteration of ALS, **a**_*f*_, **b**_*f*_, **c**_*f*_, and **d**_*f*_ are updated likewise in sequence.

Since SiPex relies only on ^15^N-HSQC, HN(CO), and ^15^N-HSQC-based relaxation spectra, some elements of the four-order tensor were missing (Fig. 2b). The missing elements were excluded from the calculation of residual errors.

In this study, we used an ALS in-house script running on MATLAB 2018b (MathWorks, MA, USA). Iterations were stopped when the relative change of the residual error between two iterations was small (less than 10^–6^) or the number of iterations reached 500. Since the ALS solution is not guaranteed as a global optimum (Bro 1997), we tried 100 ALS runs for one problem using random initial guesses and selected the solution with the smallest residual error.

### Comparison with the conventional method

For evaluation purposes, we separately obtained the main-chain assignment and relaxation values using a uniformly ^13^C/^15^N labeled protein. The main-chain assignment was achieved with standard triple resonance experiments (Ikura et al. 1990; Clubb et al. 1992; Bax and Grzesiek 1993). The R_1_, R_2_, and heteronuclear NOE data were also obtained using the uniformly labeled sample.

## Electronic supplementary material

Below is the link to the electronic supplementary material.

Supplementary file1 (PDF 5173 kb)
