# Supplementary material for: Amino-acid selective isotope labeling enables simultaneous overlapping signal decomposition and information extraction from NMR spectra
Source: J Biomol NMR. 2020 Jan 30;74(2):125–37. doi: 10.1007/s10858-019-00295-9 (PMC7080692; doi:10.1007/s10858-019-00295-9)
Supplement: Supplementary file 1 — Supplementary file1 (PDF 5173 kb) [file 10858_2019_295_MOESM1_ESM.pdf]

Supplementary Material

Amino-acid selective isotope labeling enables simultaneous overlapping signal decomposition and information extraction from NMR spectra

Journal of Biomolecular NMR

Takuma Kasai[1,2*], Shunsuke Ono[2,3], Seizo Koshiba[4,5], Masayuki Yamamoto[4,5], Toshiyuki Tanaka[6], Shiro Ikeda[7], and Takanori Kigawa[1,3*]

1. Laboratory for Cellular Structural Biology, RIKEN Center for Biosystems Dynamics Research, Yokohama, Japan

2. PRESTO, JST, Kawaguchi, Japan

3. School of Computing, Tokyo Institute of Technology, Yokohama, Japan

4. Tohoku Medical Megabank Organization, Tohoku University, Sendai, Japan

5. Graduate School of Medicine, Tohoku University, Sendai, Japan

6. Department of Systems Science, Graduate School of Informatics, Kyoto University, Kyoto, Japan

7. Department of Statistical Inference and Mathematics, The Institute of Statistical Mathematics,

Tachikawa, Japan

*Emails: takuma.kasai@riken.jp (T. Kasai); kigawa@riken.jp (T. Kigawa)

Note S1. Determination of number of components for tensor decomposition.

In this study, the number of components F was determined with decomposition trials assuming different F values. The wrong assumption of F leads to a poor explanation of the observed spectra or inconsistency with the physical backgrounds.

Fig. S1 shows the decomposition trials assuming $F = 1,\ 2$, or 3 of the ROI shown in Fig. 2. When assuming $F = 1$, there was a larger residual error (Fig. S1a) as compared to the decomposition assuming $F = 2$ (Fig. S1b, d). Moreover, the decoded amino acid pair, (H)F (Fig. S1e), did not appear in the Ub3A sequence. These results implied that the assumption $F = 1$ was not appropriate. When assuming $F = 3$, the HN(CO) intensity of component 2 of sample 2 showed a negative value (Fig. S1g), which meant a physically impossible negative isotope labeling ratio. The decoded amino acids of component 1, (K)F, did not appear in the sequence (Fig. S1g). The very small intensities for R_2 of component 1 also implied the overfitting (Fig. S1g). Considering these results, $F = 3$ was not appropriate in spite of its small residual error (Fig. S1d). Since the decomposition assuming $F = 2$ did not exhibit such overfitting (Fig. S1b, f), we concluded that $F = 2$ was the appropriate assumption. As this simulated ROI contains two overlapping signals, (I)Q62 and (R)G75, the correct answer of F is 2.

Fig. S2 shows the decomposition trials of the ROI shown in Fig. S7. The large residual error (Fig. S2a, d) and the nonexistent amino acid pair (Fig. S2e) rejected the assumption $F = 1$. When

assuming $F = 3$, the decoded amino acid pairs of components 2 and 3, (N)Q and (P)E, did not appear in the sequence. The negative elements of the loading vector along the ^{1}H dimension of component 1 also implied the overfitting (Fig. S2g). Given that $F = 2$ did not indicate overfitting (Fig. S2b, f), we concluded that $F = 2$ was the best assumption. As this ROI contains two overlapping signals, (L)E16 and (N)V26, the correct answer of F is 2.

Fig. S3 shows the decomposition trials of the ROI shown in Fig. S4a. The ^{15}N dimension is in the frequency domain. When assuming $F = 4$, there was some residual error, indicating the underestimation of F (Fig. S3a, d). Component 3 corresponded to two amide signals with the same ^{1}H chemical shift and different ^{15}N chemical shifts (Fig. S3a, e). Considering the fact that the decoded amino acid pair, (Y)V, did not exist in the sequence (Fig. S3e), F was underestimated. Assuming $F = 5$ did not indicate under- or over-estimation of F (Fig. S3b, f). As compared to the decoded amino acids assuming $F = 5$, (T)L, (G)G, (G)S, (E)D, and (M)Q (Fig. S3f), (G)G was split into two components (components 2 and 3), while the other four components were the same when assuming $F = 6$ (Fig. S3g). These two components showed similar ^{1}H and ^{15}N chemical shifts, implying that the overestimation of F led to the artificial separation of one amide signal into two components. Additionally considering that the amino acid pair (G)G appears only once in the sequence, we rejected $F = 6$ as an overestimation. In conclusion, $F = 5$ was the best assumption. As this ROI contains two overlapping signals, (G)S-2, (M)Q2, (E)D52, (T)L56, and (G)G76, the

correct answer of F is 5.

Figs. S4 and S5 show the decomposition trials of the fully-sampled (the same dataset as Fig. 3b) and NUS (the same dataset as Fig. 3c) time-domain data in the ^{15}N dimension. The residual errors when assuming $F = 4$ (Figs. S4a, d, S5a, d) implied the underestimation of F. The decoded amino acid pair (Y)V did not appear in the sequence (Figs. S4e, S5e), also implying the wrong assumption of F. Assuming $F = 6$, two components with similar ^{1}H and ^{15}N loading vectors were obtained (Figs. S4g, S5g), and both were decoded as (G)G (Figs. S4g, S5g), which appeared only once in the sequence. For similar reasons to the frequency-domain dataset (Fig. S3), we rejected the assumption $F = 6$. Since the decomposition assuming $F = 5$ did not indicate the wrong assumption, we concluded that $F = 5$ is the appropriate assumption. The correct answer of F is 5.

Note S2. Importance of selectively labeled samples for tensor decomposition.

The NMR spectra acquired with SiCode samples can be regarded as a four-order tensor in SiPex (Eq. 9), while those acquired with a uniformly labeled sample can be regarded as a three-order tensor in relaxation measurement by MUNIN (Eq. 2) (Korzhnev et al. 2001). The additional dimension, SiCode, not only provides amino-acid information but also facilitates the decomposition of overlapping signals. In this supplementary note, we discuss the improvements of the decomposition by the additional SiCode dimension.

The two-dimensional spectra acquired with a uniformly $^{13}C/^{15}N$-labeled sample were assembled to form the three-order tensor illustrated in Fig. S8a. Hereafter, we refer to the decomposition of this three-order tensor as the "MUNIN-equivalent". The relaxation measurements utilized for the MUNIN-equivalent, R_1, R_2, and NOE, are different from those utilized in the original MUNIN relaxation measurements, $R_1\rho$ (Korzhnev et al. 2001). In spite of the difference, the tensor can be decomposed to loading vectors in the same way as the original MUNIN relaxation measurement, according to Eq. 2.

Fig. S8b shows the MUNIN-equivalent of the same simulated ROI, which contains artificial overlapping signals, (I)Q62 and (R)G75, as in Fig. 2. In this case, the MUNIN-equivalent successfully decomposed the two overlapping signals (Fig. S8b) to extract the R_1, R_2, and NOE values, which were in good agreement with those obtained from the conventional methods (Table 1).

The assignment of the components to the residues is based only on the slight differences in the chemical shifts (Fig. S8b). Note that, in contrast, the additional amino-acid information greatly facilitates the assignments in SiPex (Figs. 2d, S1f).

The MUNIN-equivalent of the same ROI as in Fig. S7, containing actual overlapping signals (L)E16 and (N)V26, was unsuccessful (Fig. S9a, b). The loading vectors of the two components along the ^{1}H dimension had both positive and negative values, which is less likely on the phase-corrected NMR spectra. This failure is probably due to the similar loading vectors of the two components along the relaxation dimension (Fig. S2f). As noted in the main text, the other two dimensions are insufficient for a unique solution, due to rotational ambiguity (Orekhov et al. 2001). In contrast, the decomposition by SiPex was successful, because there were still three remaining dimensions, ^{1}H, ^{15}N, and SiCode (Fig. S2f).

Some tensor decomposition software programs apply constraints, such as non-negativity and unimodality, to the loading vectors. The non-negativity constraint on the loading vectors on all dimensions prevents the appearance of negative signals, and as a consequence avoids the decomposition failure described above. Fig. S9c, d shows the successful decomposition of the same dataset as in Fig. S9a, b when non-negativity constraints were applied. In this case, the application of the non-negativity constraints is reasonable, because the NOE enhancements of these non-terminal residues of the globular protein are expected to be positive. However, in general, the non-negativity

constraints on the relaxation dimension are not always applicable, since the signals on heteronuclear NOE spectra may be negative. The decomposition failed when the non-negativity constraints were applied only on the ^{1}H and ^{15}N dimensions (Fig. S9e, f). More flexible constraints, such as applying non-negativity on some elements of the loading vector, should help the decomposition in these cases. We have proposed a tensor decomposition program that is compatible with such flexible constraints (Ono and Kasai 2018).

We also performed the MUNIN-equivalent of the same ROI shown in Fig. 3. In the decomposition of the ^{15}N frequency-domain data, each component contained multiple signals, indicating decomposition failure (Fig. S10a, b). This is because the loading vectors of some signals along the ^{1}H and relaxation dimensions are similar (Fig. S3b, f). Applying non-negativity (Fig. S10c) or unimodality (Fig. S10d) constraints on the ^{1}H and ^{15}N dimensions did not resolve the mixing problem, because non-negativity was not applied to the relaxation dimension for the aforementioned reason. The signals were also mixed in the ^{15}N fully-sampled time-domain data (Fig. S11a) and the NUS time-domain data (Fig. S11b), indicated by the loading vectors along the ^{1}H dimension. Note that non-negativity or unimodality constraints are not applicable to the time-domain dimension.

In the original MUNIN relaxation measurement, the relaxation dimension is an assembly of $R_{1}\rho$ relaxation curves at different spin-lock offsets (Korzhnev et al. 2001), and thus the difference of the relaxation rate dependence on the spin-lock offsets of the overlapping signals facilitates the

decomposition. It is important to ensure at least three dimensions with different loading vectors for the decomposition without signal mixing (Orekhov et al. 2001). The SiCode dimension makes SiPex more robust than the MUNIN-equivalent in cases where the loading vectors of one of the dimensions are virtually identical, as demonstrated by the successful signal decomposition by SiPex (Figs. 3, S2b, f, S3b, f, S4b, f, S5b, f, S7), in contrast to the MUNIN-equivalent (Figs. S9a, b, S10a, b, S11a, b).

Note S3. Tensor decomposition with a publicly available program.

In this paper, we used a simple, in-house ALS program to solve the PD problem (See Materials and Methods). The concept of SiPex, to extract information from the loading vectors as a result of PD, is independent from the method of tensor decomposition. Therefore, various other programs/algorithms that can solve PD are also applicable. For example, alternating optimization with primal-dual splitting (Ono and Kasai 2018), a faster algorithm compatible with various regularizations, may be more suitable.

One of the publicly available programs that can solve PD is ‘N-way toolbox’ (Andersson and Bro 2000). It solves PD using ALS implemented with various acceleration methods (Bro 1997). We confirmed that the same results as our in-house program were obtained with N-way toolbox (Fig. S14) with ‘random orthogonalized values’ as the initialization option. The computation times to decompose the datasets shown in Fig. S14a-e with 100 random initializations were 1.8, 1.3, 78, 59, and 15 seconds, respectively, using dual Xeon E5-2690 v4 CPUs. The corresponding computation times using the in-house program were 27, 26, 422, 399, and 38 seconds, respectively. Therefore, we recommend the publicly available programs such as N-way toolbox for practical use.

Note S4. Compensation of concentration differences between samples.

Since amino-acid discrimination by SiCode and SiPex depends on the signal intensity ratios of the samples, it is important to compensate for systematic errors between samples (named “intensity disturbances” in the previous report (Kasai et al. 2015)) derived mainly from the differences of sample concentrations (Kasai et al. 2015). The sample concentrations can be estimated from the observed signal intensities using a modified method of those described in Kasai et al. (2015). Let i be the index of a signal on the ^{15}N-HSQC spectrum, A_i the signal intensity of the i-th signal when the sample is fully ^{15}N labeled, a_i an amino acid of the i-th signal, j an index of the labeled sample, c_j the concentration of the j-th sample, $n_j(a)$ the ^{15}N labeling ratio of the j-th sample for the amino acid a. The sum of the squared errors E to the observed intensities of the i-th signal of the j-th sample, I_{ij}, is

$$E = \sum_i \sum_j \left(I_{ij} - c_j A_i n_j(a_i)\right)^2$$

We estimated unknown variables, c_j, A_i, and a_i, to minimize E with a simulated annealing method. For the purpose of concentration estimation, overlapping signals should be excluded because their intensities may contain errors. Since the selection of “isolated signals” is based on visual inspection, they may contain a small number of overlapping signals, which minimally affect the overall estimation of concentrations. After the concentration estimation with the ^{15}N-HSQC spectrum, all of the spectra (^{15}N-HSQC, HN(CO), ^{15}N-R$_1$-HSQC, ^{15}N-R$_2$-HSQC, and ^{1}H-^{15}N

heteronuclear NOE-HSQC) were divided by the estimated values c_j prior to the tensor decomposition analysis. For Ub3A analysis, 72 “isolated” signals were selected. The estimated c_j values for 3 samples were 1.00, 0.94, and 0.93. For Nrf2 Neh2 analysis, 23 “isolated” signals were selected. The estimated c_j values for 4 samples were 0.87, 0.90, 0.94, and 1.14.

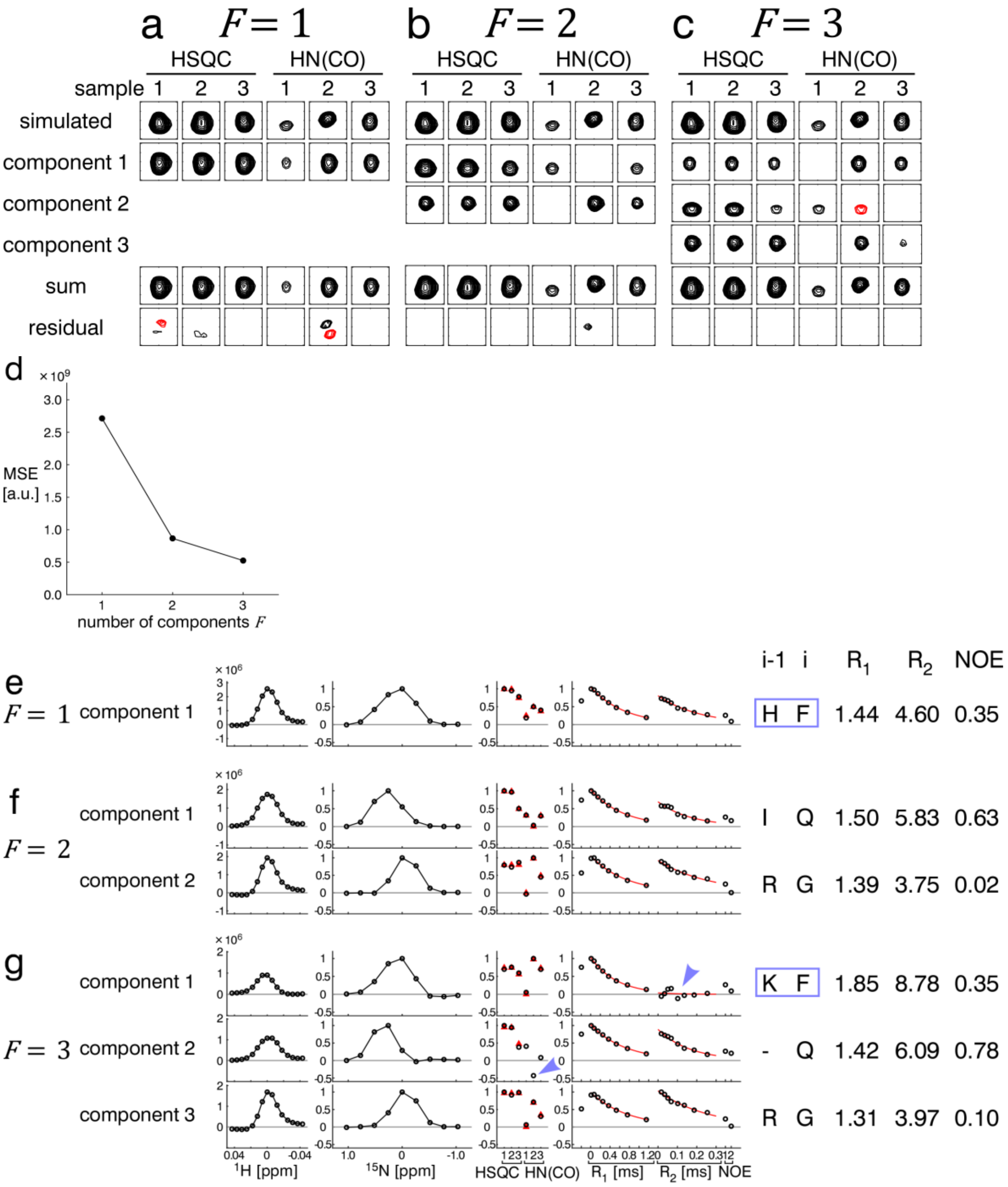

Fig. S1. Trials of decomposition of simulated overlapping signals (I)Q62 and (R)G75 with different numbers of components.

(a-c) Decomposition of the dataset shown in Figure 2, assuming that the number of components F is (a) 1, (b) 2, and (c) 3. The simulated spectra with signal overlapping, the reconstructed spectra

from the loading vectors of the decomposed components, the sum of the reconstructed spectra, and the residual error are shown from the top to the bottom. Only HSQC and HN(CO) are presented. Black and red lines are positive and negative contours, respectively. (d) Mean square errors (MSEs) of the decomposition plotted against F. (e-g) The loading vectors and the extracted information of decompositions, assuming F is (e) 1, (f) 2, and (g) 3. The loading vectors of four dimensions, ^{1}H ($\mathbf{a}_f$), ^{15}N ($\mathbf{b}_f$), SiCode ($\mathbf{c}_f$), and relaxation ($\mathbf{d}_f$), are plotted from left to right. Black circles and lines are the loading vectors. Red triangles indicate best-fit parameters for decoding amino-acid information. Red lines indicate exponential fitting for the extraction of relaxation properties. On the right of the plots, the extracted information, the amino acids at residues i-1 and i, the relaxation constants of R_1 and R_2 (in s^{-1}), and the NOE enhancements, is shown. Blue arrowheads and squares indicate negative elements of the loading vectors and inconsistent amino acids, respectively, which imply the wrong assumption of F.

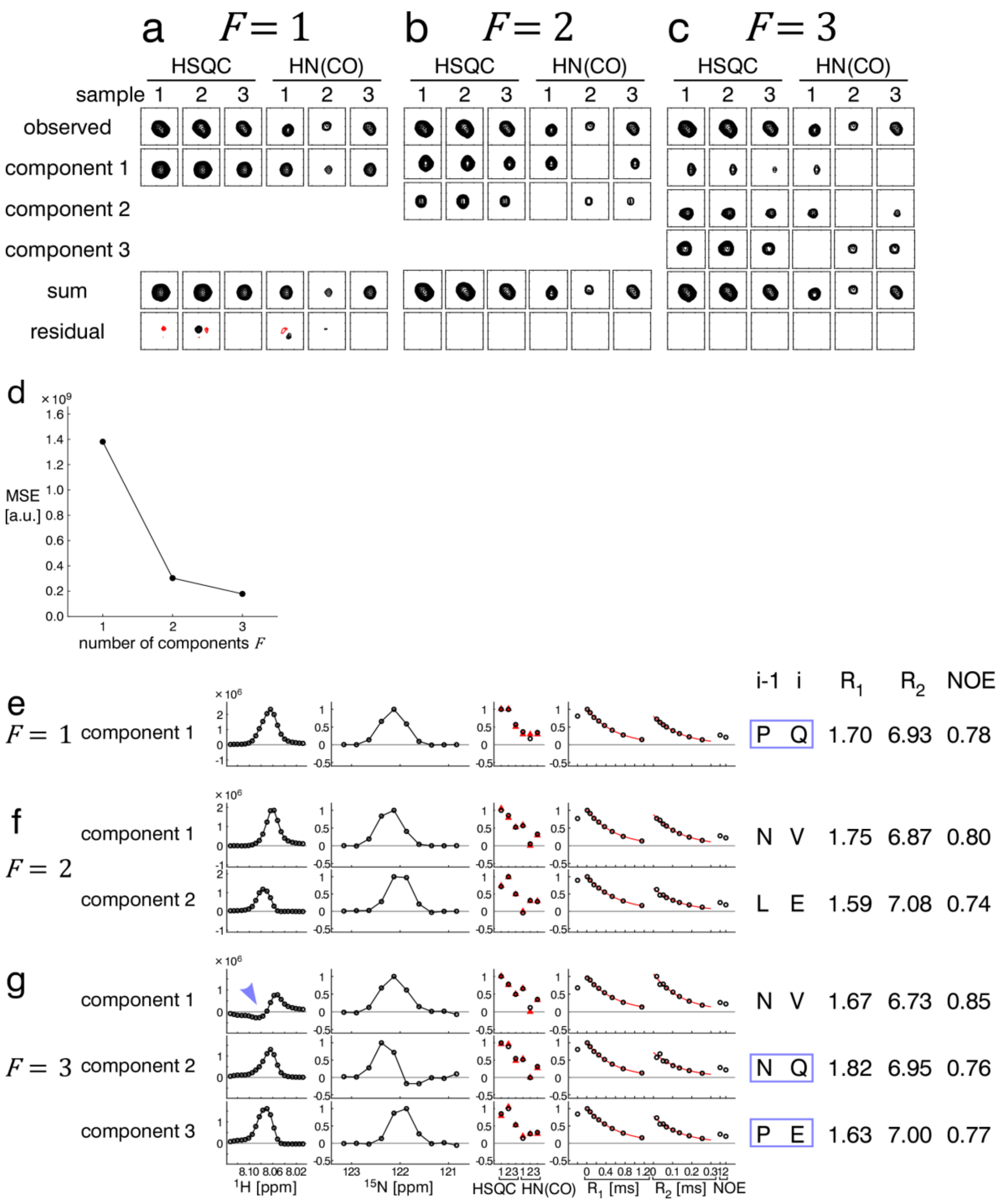

Fig. S2. Trials of decomposition of actual overlapping signals (L)E16 and (N)V26 with different numbers of components.

Decompositions of the dataset shown in Fig. S7 assuming $F = 1$ to 3 are presented as in Fig. S1.

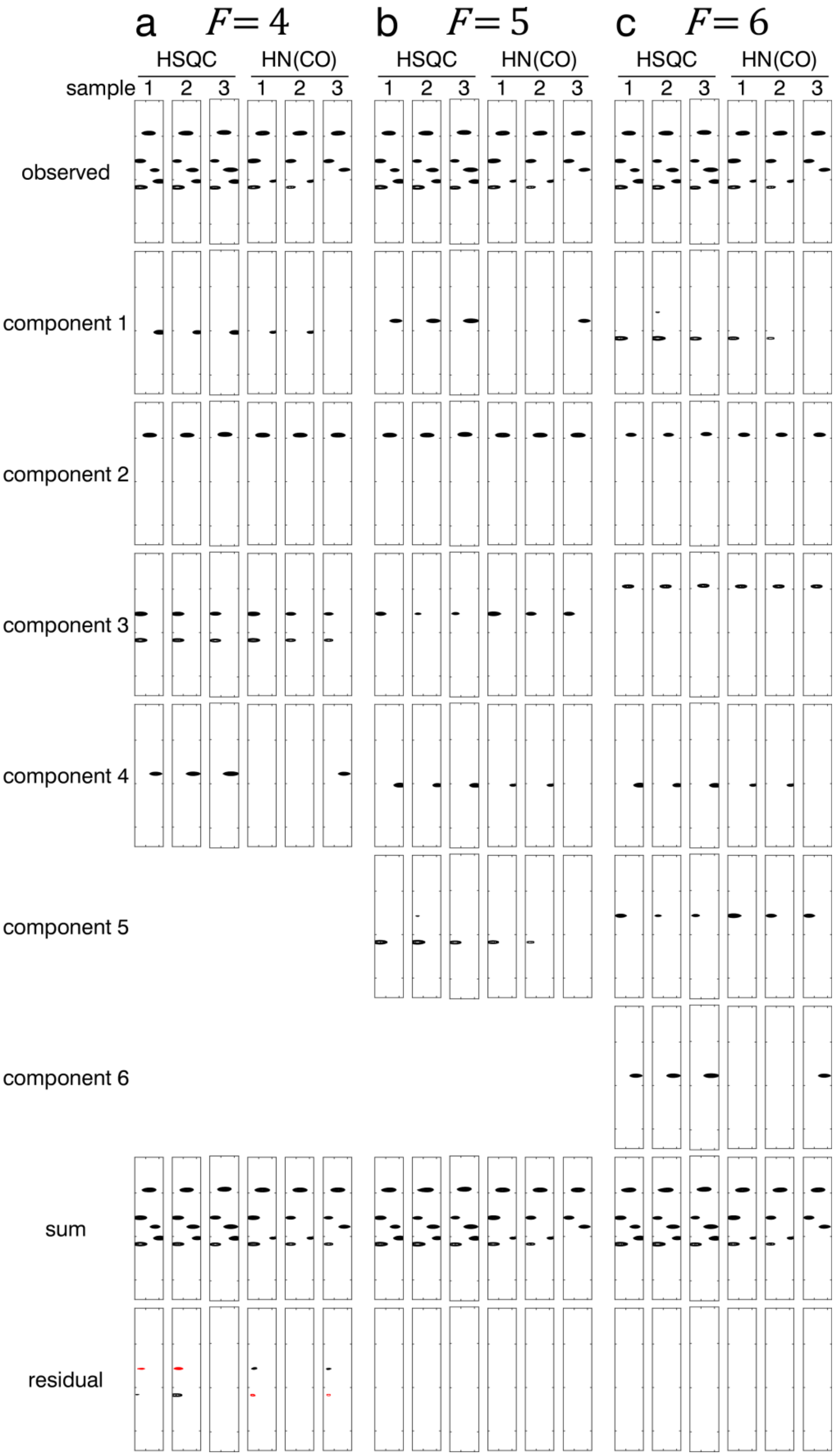

a F = 4
b F = 5
c F = 6
HSQC
HN(CO)
sample 1 2 3 1 2 3
observed
component 1
component 2
component 3
component 4
component 5
component 6
sum
residual

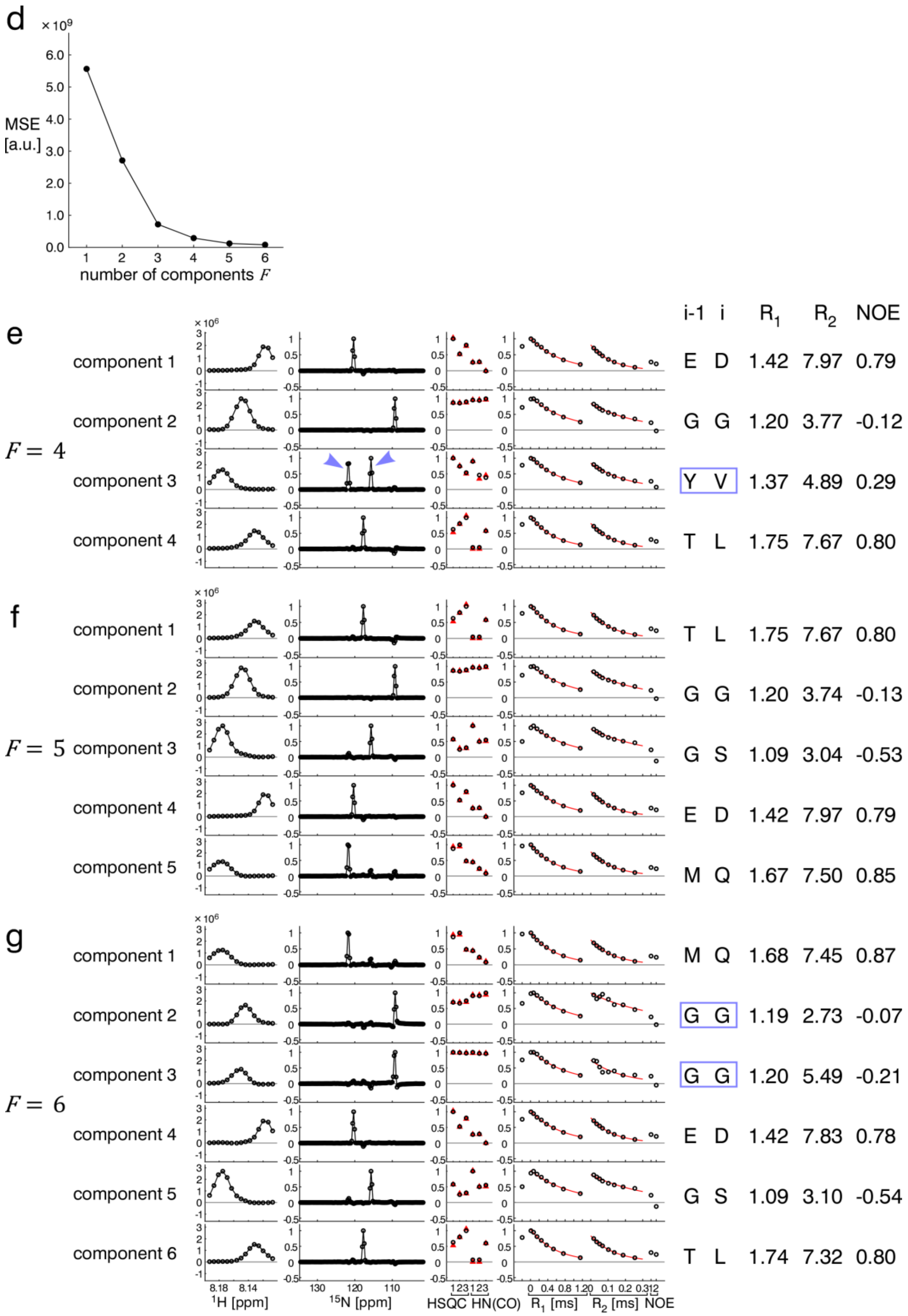

Fig. S3. Trials of decomposition of frequency-domain dataset with different numbers of

components.

Decompositions of the dataset shown in Fig. 3a assuming $F = 4$ to 6 are presented as in Fig. S1.

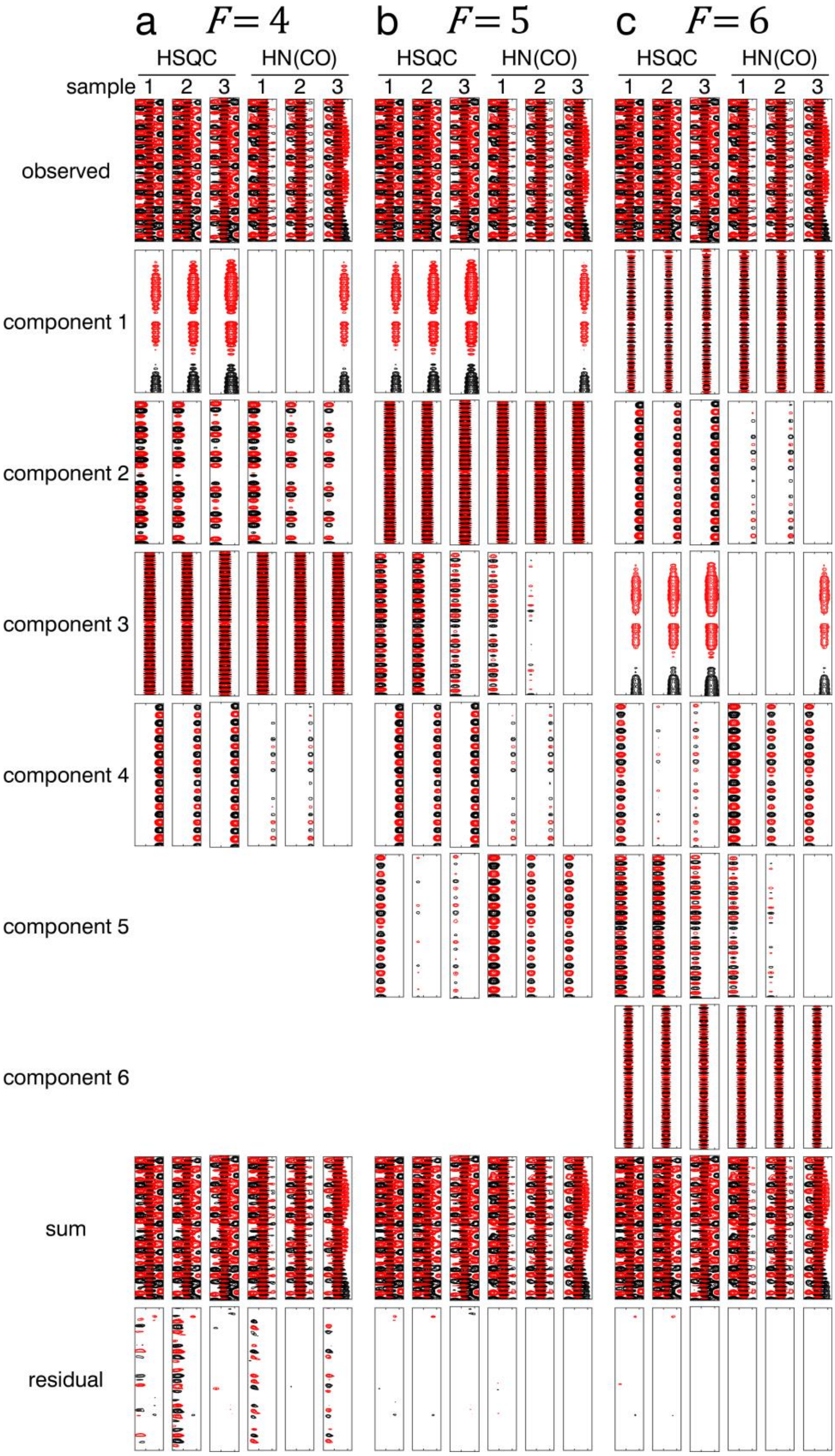

a F = 4
b F = 5
c F = 6
HSQC
HN(CO)
sample
1
2
3
observed
component 1
component 2
component 3
component 4
component 5
component 6
sum
residual

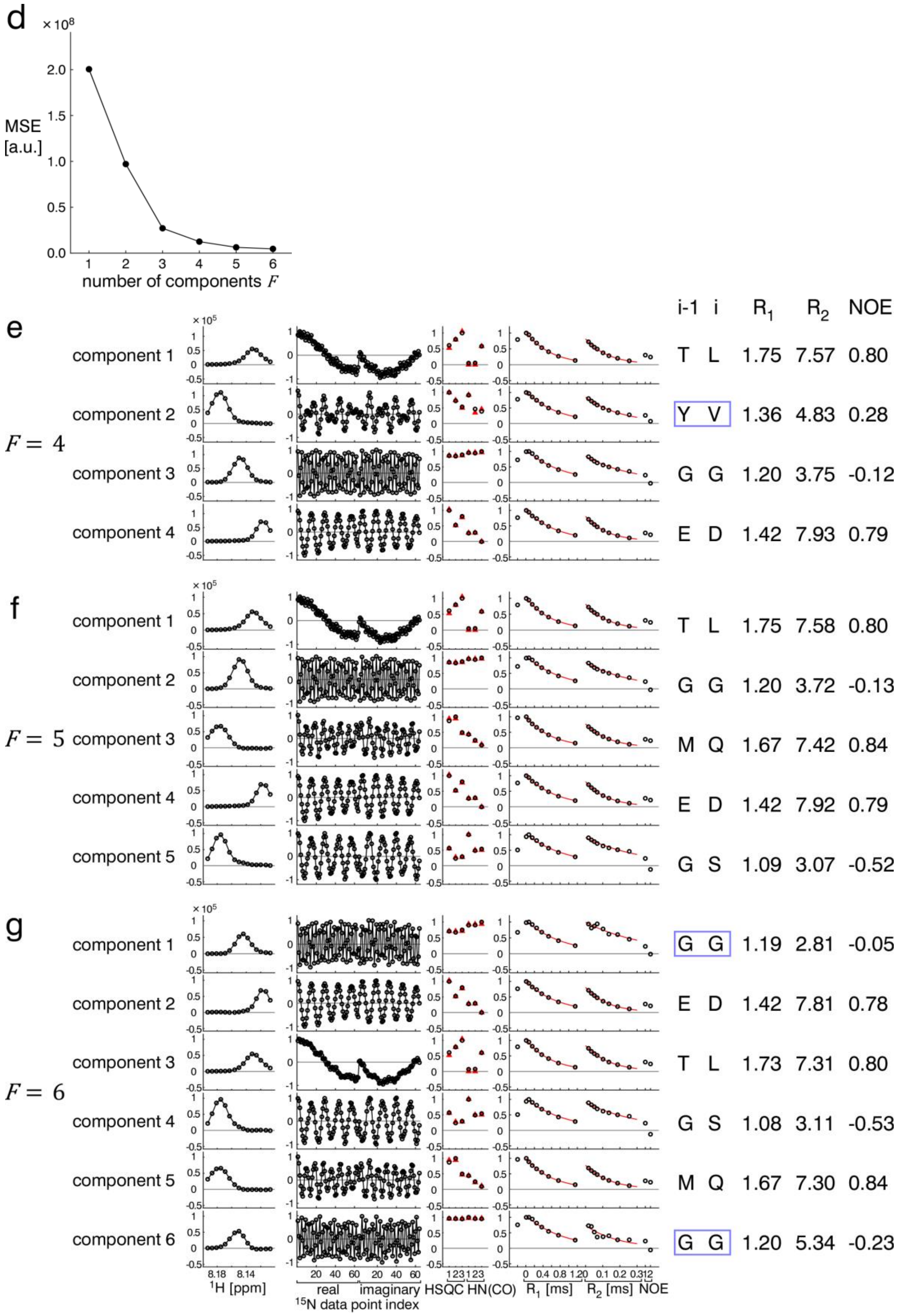

Fig. S4. Trials of decomposition of fully-sampled time-domain dataset with different numbers

of components.

Decompositions of the dataset shown in Fig. 3b assuming $F = 4$ to 6 are presented as in Fig. S1.

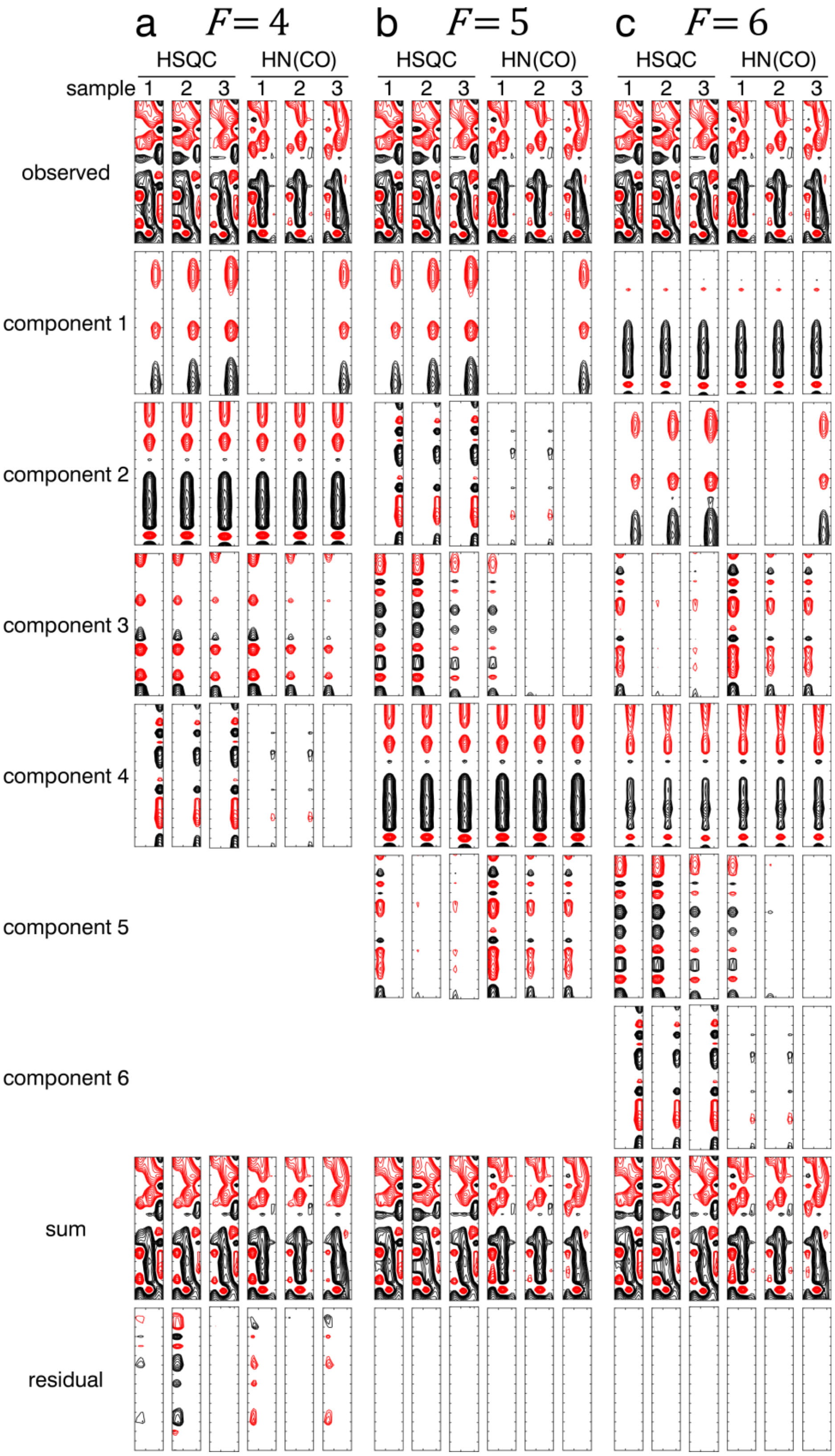

a $F = 4$
b $F = 5$
c $F = 6$
HSQC
HN(CO)
sample 1 2 3 1 2 3
observed
component 1
component 2
component 3
component 4
component 5
component 6
sum
residual

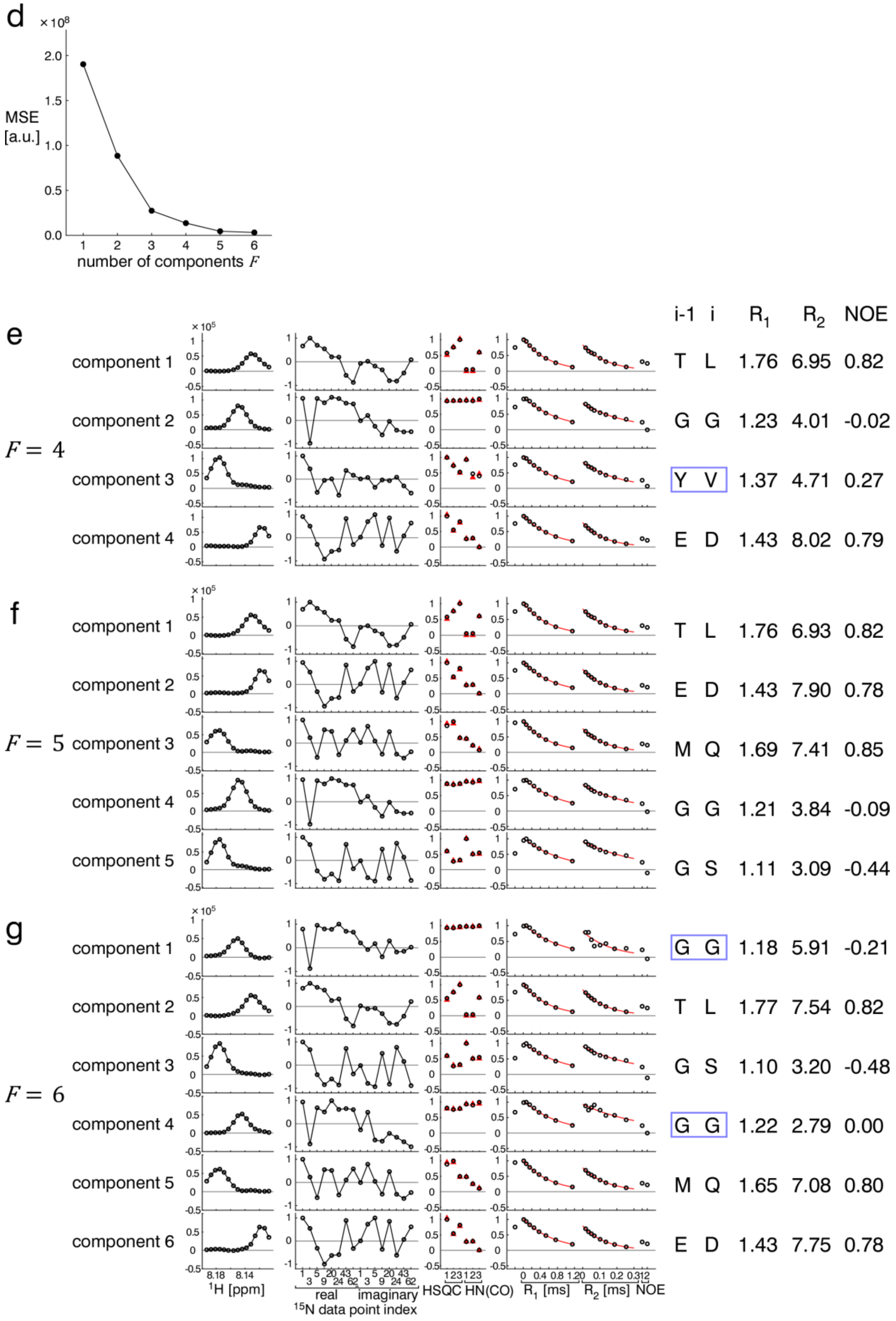
d
MSE [a.u.]
×10^8
number of components F
e
F = 4
f
F = 5
g
F = 6
component 1
component 2
component 3
component 4
component 5
component 6
1H [ppm]
real
imaginary
15N data point index
HSQC HN(CO)
R1 [ms]
R2 [ms]
NOE
i-1 i R1 R2 NOE
T L 1.76 6.95 0.82
G G 1.23 4.01 -0.02
Y V 1.37 4.71 0.27
E D 1.43 8.02 0.79
T L 1.76 6.93 0.82
E D 1.43 7.90 0.78
M Q 1.69 7.41 0.85
G G 1.21 3.84 -0.09
G S 1.11 3.09 -0.44
G G 1.18 5.91 -0.21
T L 1.77 7.54 0.82
G S 1.10 3.20 -0.48
G G 1.22 2.79 0.00
M Q 1.65 7.08 0.80
E D 1.43 7.75 0.78

Fig. S5. Trials of decomposition of NUS time-domain dataset with different numbers of components.

Decompositions of the dataset shown in Fig. 3c assuming $F = 4$ to 6 are presented as in Fig. S1.

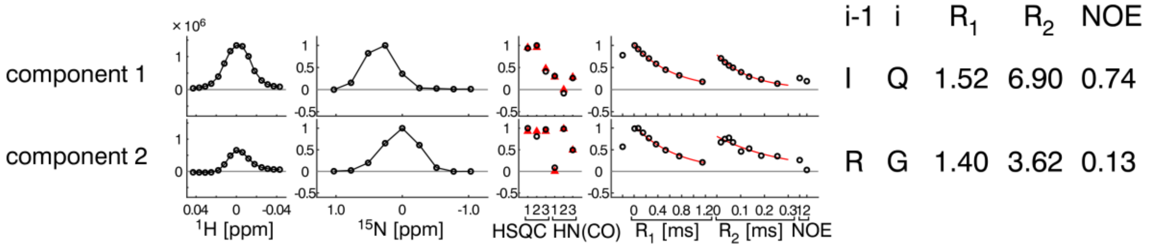

Fig. S6. Decomposition of simulated overlapping signals with different intensity levels.

The same experiment as Fig. 2 but the ROI of (R)G75 was multiplied by 0.3 prior to the merging of 2 ROIs.

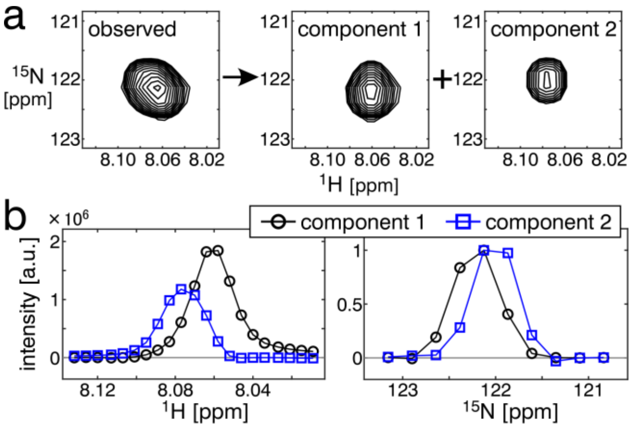

Fig. S7. Decomposition of actual overlapping signals.

(a) The ROI of the observed spectrum containing the (L)E16 and (N)V26 signals (left panel) was decomposed into two components (right two panels). Only the ^{15}N HSQC spectrum of sample 1 is shown. (b) Loading vectors along the ^{1}H (left) and ^{15}N (right) dimensions. The markers and line styles are the same as in Fig. 2c.

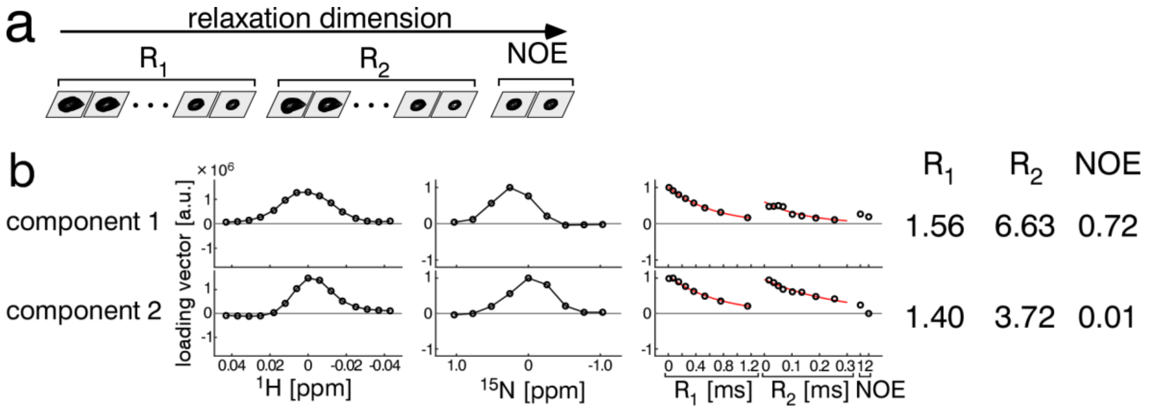

Fig. S8. Decomposition of a three-order tensor with a uniformly labeled sample (MUNIN-equivalent) of simulated overlapping signals (I)Q62 and (R)G75.

(a) Illustration of three-order tensor formation with a set of 2D spectra in the MUNIN-equivalent. (b) The loading vectors and the extracted relaxation information by the MUNIN-equivalent. The loading vectors of three dimensions, ^{1}H, ^{15}N, and relaxation, are plotted from left to right. Black circles and lines are the loading vectors. Red lines indicate exponential fitting for the extraction of relaxation properties. On the right of the plots, the extracted information, the relaxation constants of R_1 and R_2 (in s^{-1}) and the NOE enhancements, is shown.

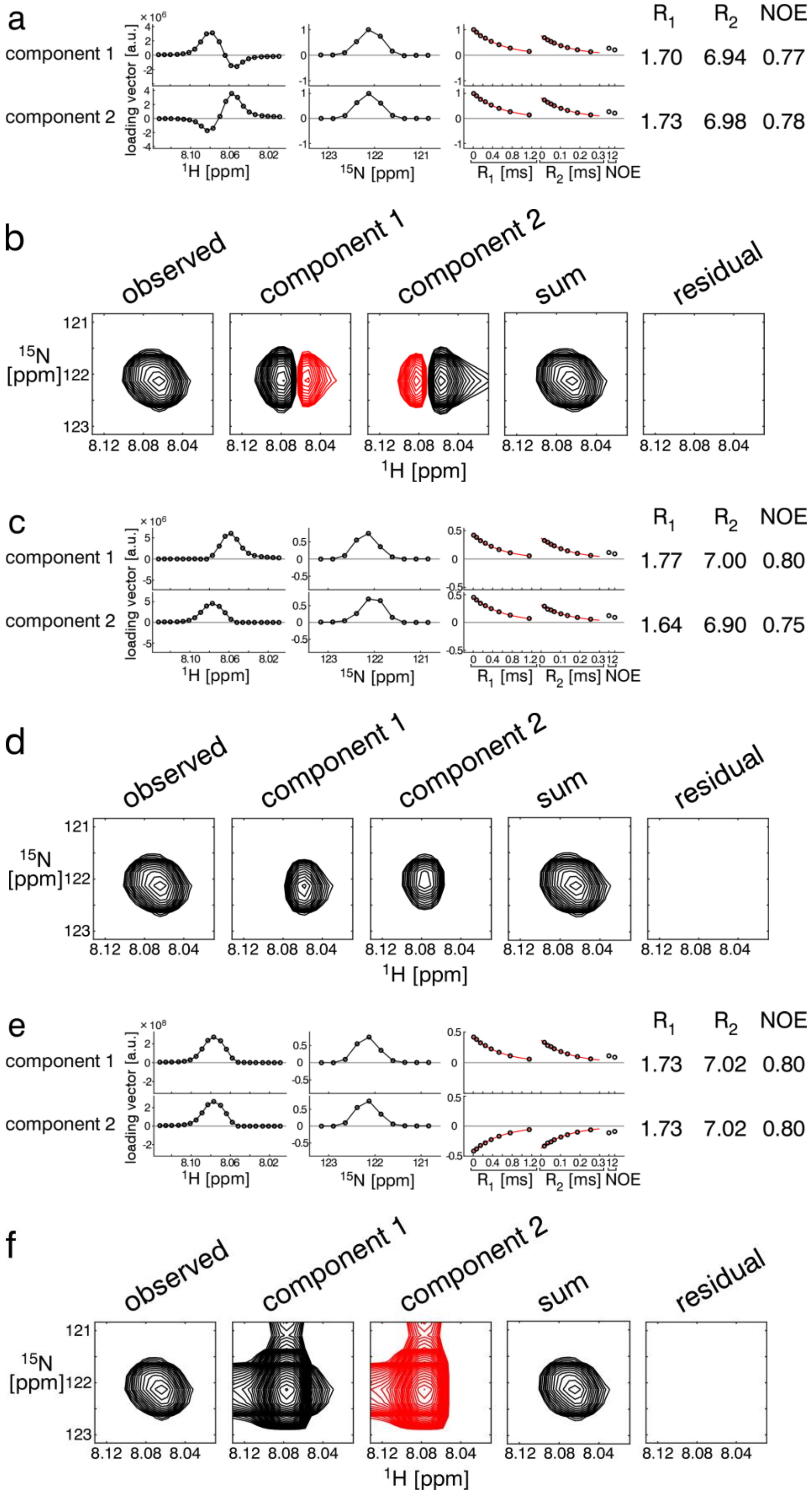

a
component 1
component 2
loading vector [a.u.]
^{1}H [ppm]
^{15}N [ppm]
R_1 [ms] R_2 [ms] NOE
R_1 R_2 NOE
1.70 6.94 0.77
1.73 6.98 0.78
b
observed
component 1
component 2
sum
residual
^{15}N [ppm]
^{1}H [ppm]
c
component 1
component 2
loading vector [a.u.]
^{1}H [ppm]
^{15}N [ppm]
R_1 [ms] R_2 [ms] NOE
R_1 R_2 NOE
1.77 7.00 0.80
1.64 6.90 0.75
d
observed
component 1
component 2
sum
residual
^{15}N [ppm]
^{1}H [ppm]
e
component 1
component 2
loading vector [a.u.]
^{1}H [ppm]
^{15}N [ppm]
R_1 [ms] R_2 [ms] NOE
R_1 R_2 NOE
1.73 7.02 0.80
1.73 7.02 0.80
f
observed
component 1
component 2
sum
residual
^{15}N [ppm]
^{1}H [ppm]

Fig. S9. Decomposition of a three-order tensor with a uniformly labeled sample (MUNIN-equivalent) of actual overlapping signals (L)E16 and (N)V26.

(a, b) Decomposition without any constraints, using an in-house ALS program. (c, d) Decomposition with non-negativity constraints to all three dimensions, ^{1}H, ^{15}N, and relaxation, using the N-way toolbox program (Andersson and Bro 2000). (e, f) Decomposition with non-negativity constraints to only two dimensions, ^{1}H and ^{15}N, using the N-way toolbox program (Andersson and Bro 2000). (a, c, e) The loading vectors and extracted relaxation information are presented as in Fig. S9b. (b, d, f) The observed spectrum, the reconstructed spectra from the loading vectors of the decomposed components, the sum of the reconstructed spectra, and the residual error are shown from left to right. Only the first point for the relaxation delay of R_1-HSQC is presented. Black and red lines are positive and negative contours, respectively.

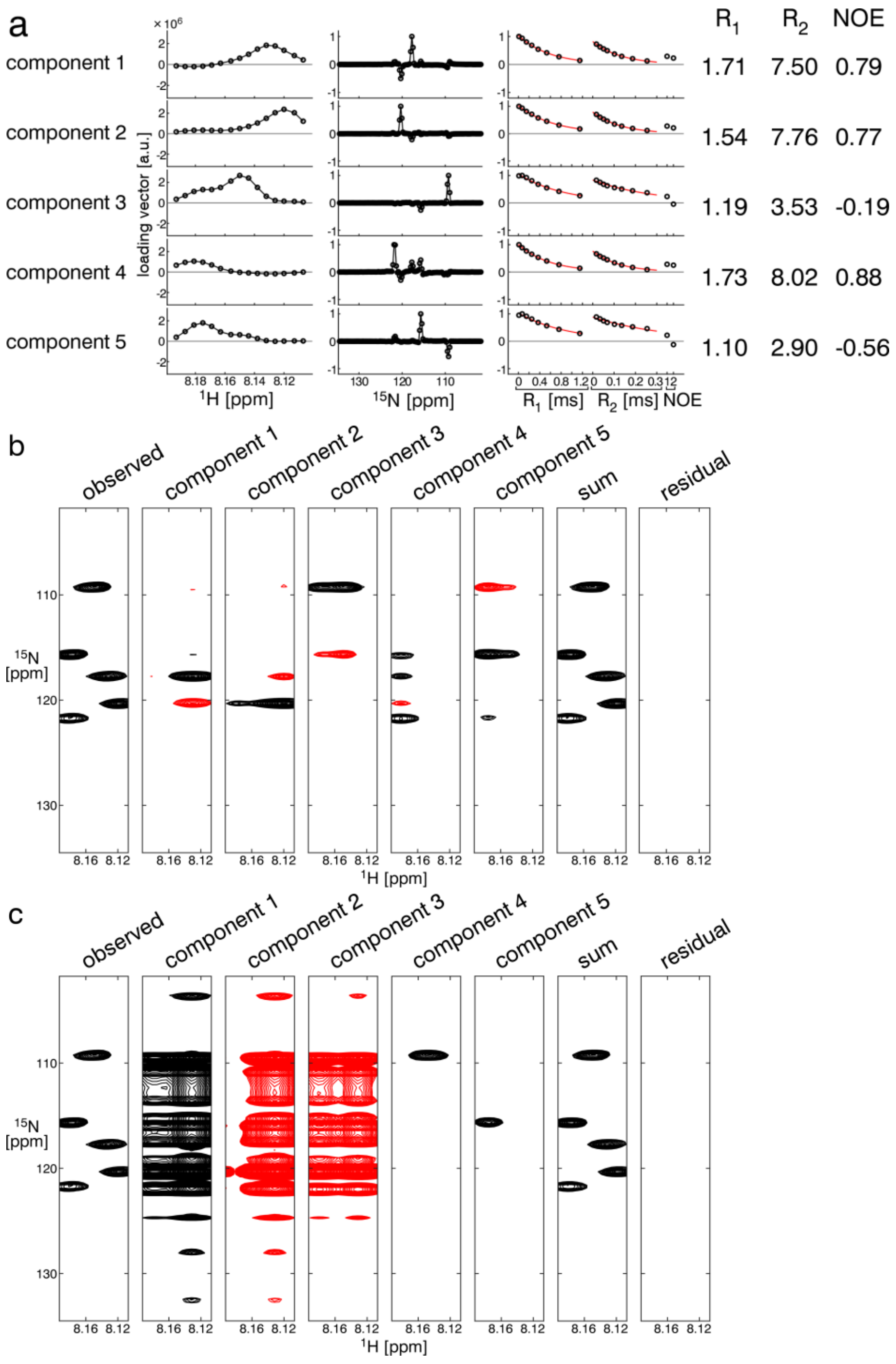
a
component 1
component 2
component 3
component 4
component 5
loading vector [a.u.]
×10^6
R1 R2 NOE
1.71 7.50 0.79
1.54 7.76 0.77
1.19 3.53 -0.19
1.73 8.02 0.88
1.10 2.90 -0.56
1H [ppm]
15N [ppm]
R1 [ms] R2 [ms] NOE
b
observed
component 1
component 2
component 3
component 4
component 5
sum
residual
15N [ppm]
1H [ppm]
c
observed
component 1
component 2
component 3
component 4
component 5
sum
residual
15N [ppm]
1H [ppm]

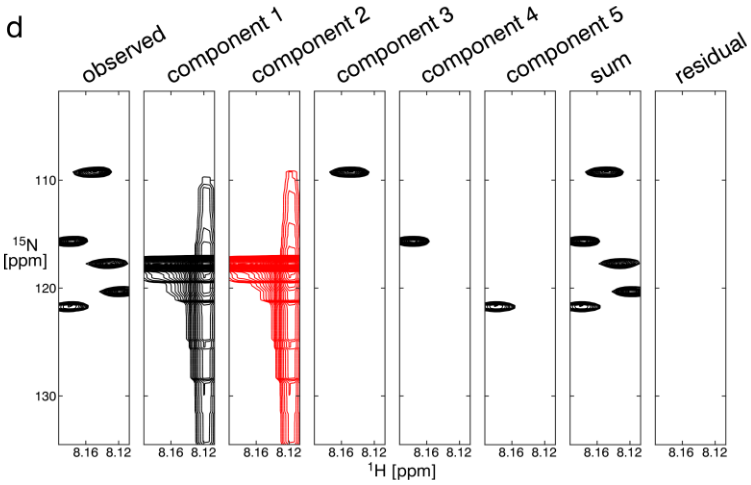

Fig. S10. Decomposition of a three-order tensor with a uniformly labeled sample (MUNIN-equivalent) of the frequency-domain dataset.

(a, b) Decomposition without any constraints, using the in-house ALS program. (c) Decomposition with non-negativity constraints to the ^{1}H and ^{15}N dimensions using the N-way toolbox program (Andersson and Bro 2000). (d) Decomposition with unimodality constraints to the ^{1}H and ^{15}N dimensions, using the N-way toolbox program (Andersson and Bro 2000). (a) The loading vectors and extracted relaxation information are presented as in Fig. S9b. (b, c, d) The observed, reconstructed, sum, and residual spectra are presented as in Fig. S10b.

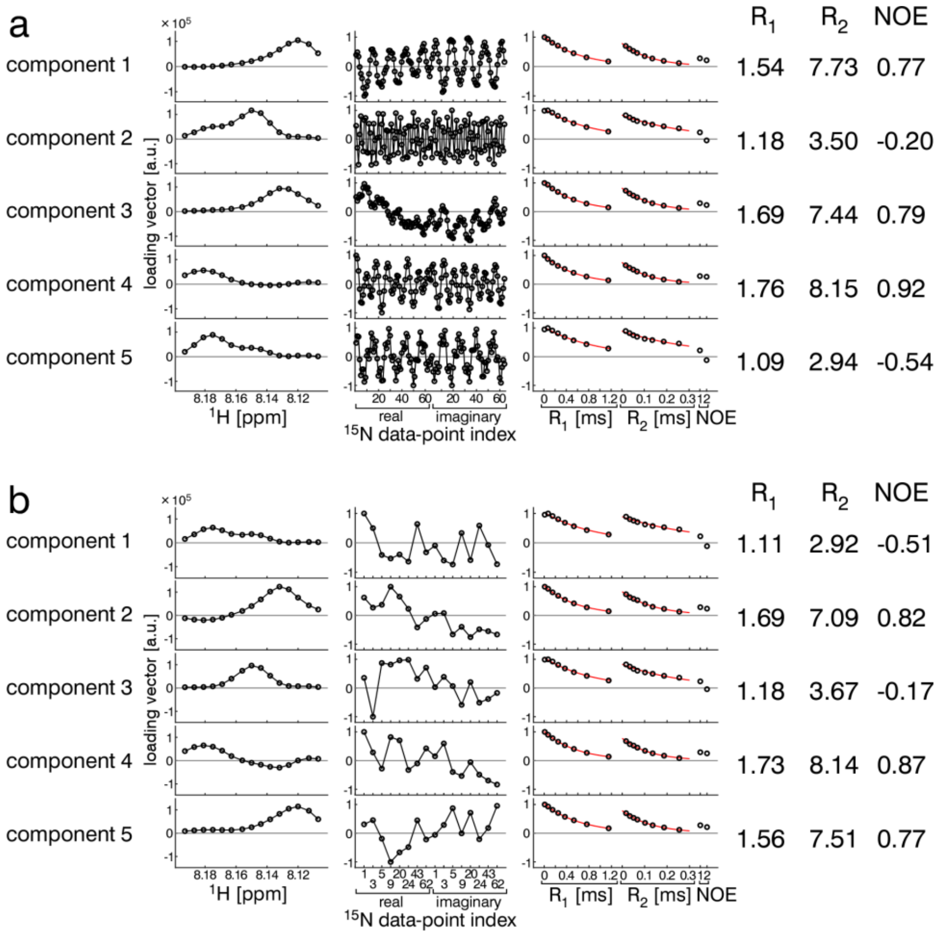

Fig. S11. Decomposition of a three-order tensor with a uniformly labeled sample (MUNIN-equivalent) of the ^{15}N time-domain dataset.

(a) Decomposition of the fully-sampled time-domain dataset without any constraints, using the in-house ALS program. (b) Decomposition of the NUS time-domain dataset without any constraints, using the in-house ALS program. (a,b) The loading vectors and extracted relaxation information are presented as in Fig. S9b.

amino acid	sample 1		sample 2		sample 3		sample 4	
	^{13}C	^{15}N	^{13}C	^{15}N	^{13}C	^{15}N	^{13}C	^{15}N
G	100	100	100	100	100	100	100	100
F	100	100	100	100	50	75	50	75
P	100	100	100	100	0	0	0	0
L	100	100	50	75	100	100	0	50
S	100	100	50	75	50	75	100	100
D	100	100	50	75	0	50	0	50
M	100	100	0	50	100	100	100	100
K	100	100	0	50	50	75	50	75
R	100	100	0	50	0	50	100	100
A	50	75	100	100	100	100	0	50
I	50	75	100	100	50	75	100	100
V	50	75	50	75	100	100	50	75
Y	50	75	0	50	100	100	0	50
Q	0	50	100	100	100	100	100	100
E	0	50	100	100	50	75	0	50
H	0	50	100	100	0	50	100	100
T	0	50	50	75	100	100	0	50
W	0	50	0	50	100	100	100	100

● 100% ^{15}N ◕ 75% ^{15}N ◑ 50% ^{15}N

● 100% ^{13}C ◑ 50% ^{13}C ○ 0% ^{13}C

Fig. S12. The labeling pattern used in this study for Nrf2 Neh2 domain.

The ^{13}C and ^{15}N labeling ratios are indicated as percentages.

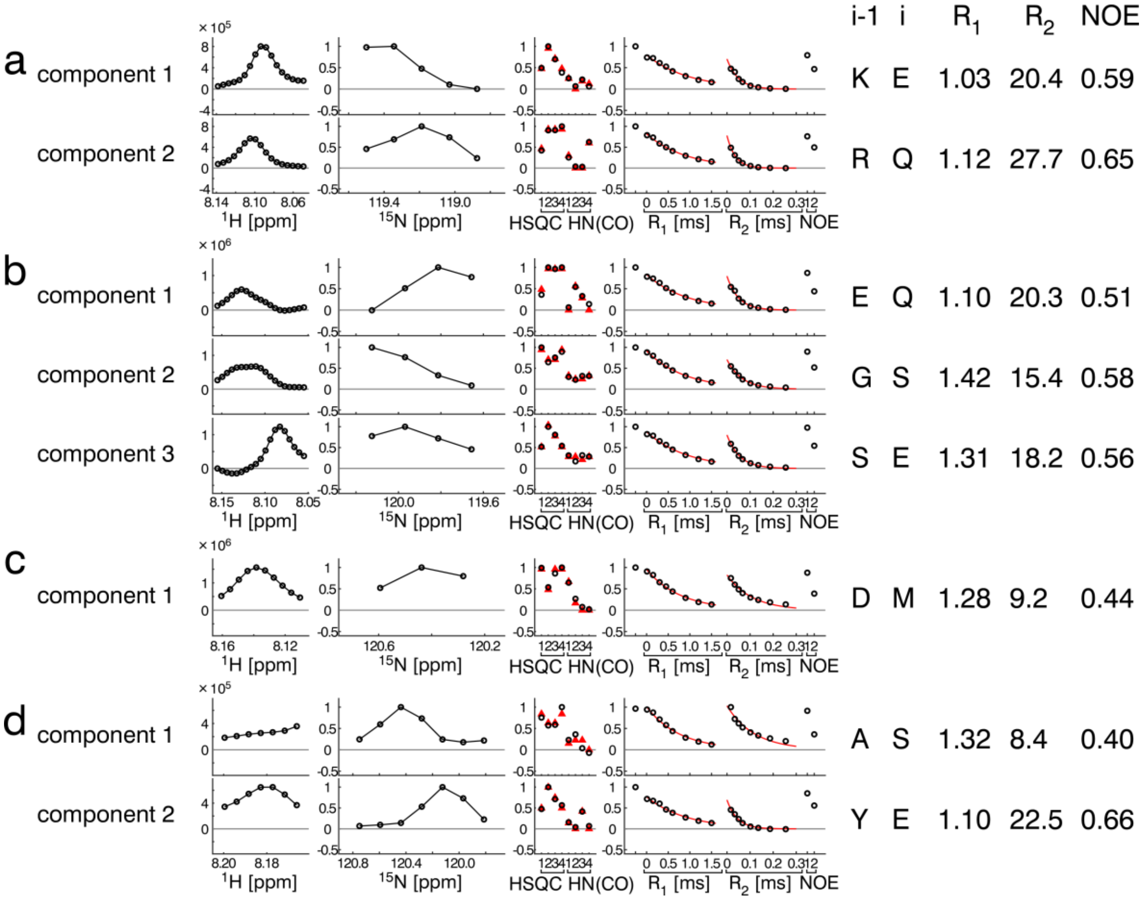

Fig. S13. Decomposition of a crowded spectral region of Nrf2 Neh2 domain.

Decomposition of the spectral region shown in Fig. 4a. Panels (a-d) correspond to subregions 1-4 in Fig. 4a, respectively. The loading vectors, and the amino-acid and relaxation information are shown.

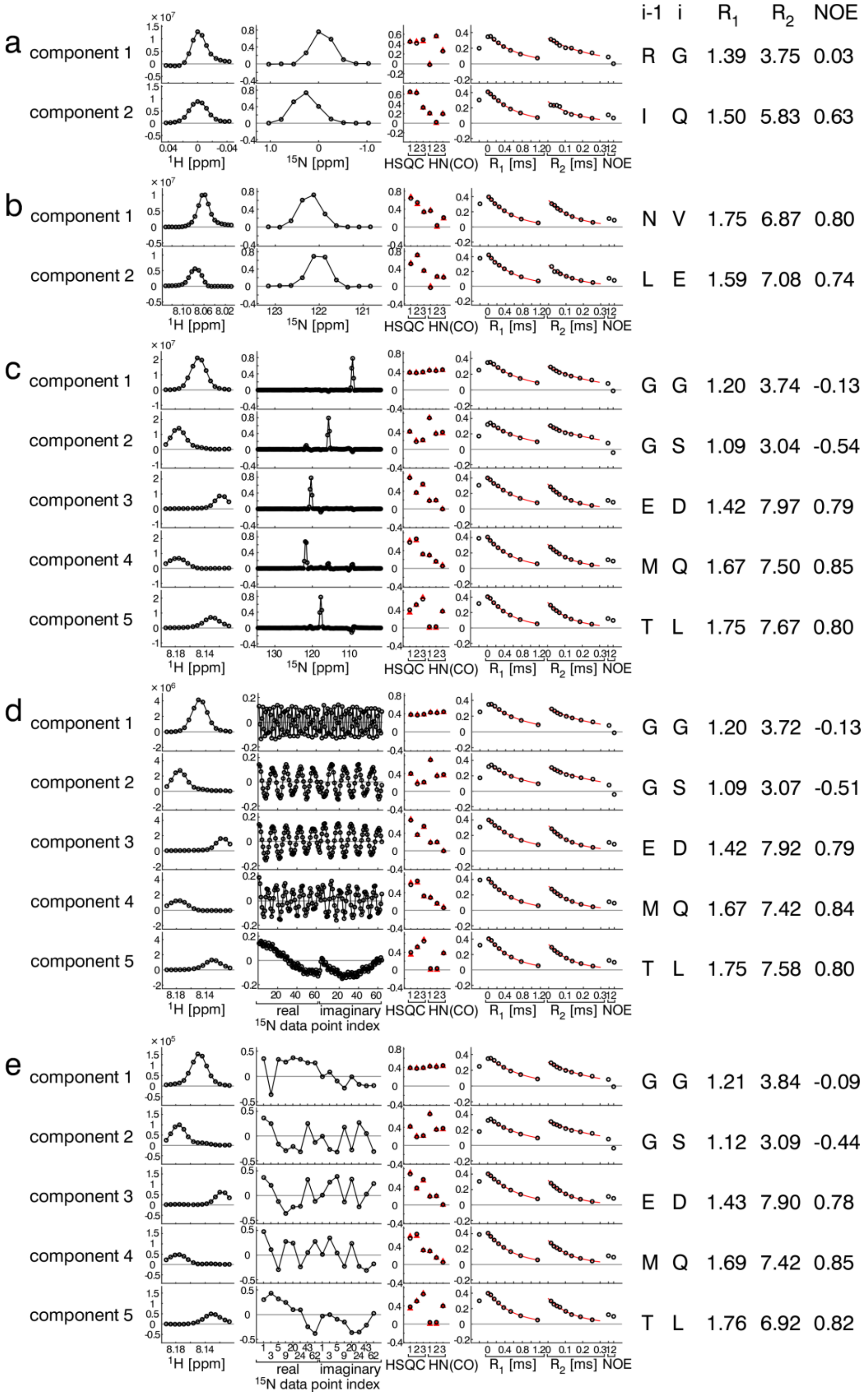

Fig. S14. Decomposition with a publicly available program.

The same datasets as (a) Fig. S1f, (b) Fig. S2f, (c) Fig. S3f, (d) Fig. S4f, and (e) Fig. S5f were decomposed using the ‘N-way toolbox’ program (Andersson and Bro 2000).

Table S1 Compositions[a] of amino acid mixture solutions for cell-free synthesis of Ub3A

Amino acid	for Sample 1			for Sample 2			for Sample 3		
	NL[b]	^{15}N	$^{13}C/^{15}N$	NL[b]	^{15}N	$^{13}C/^{15}N$	NL[b]	^{15}N	$^{13}C/^{15}N$
G	0	0	7.5	0	0	7.5	0	0	7.5
F	0	0	7.5	0	0	7.5	1.875	1.875	3.75
Q	0	0	7.5	0	0	7.5	3.75	3.75	0
Y	0	0	7.5	1.875	1.875	3.75	0	0	7.5
M	0	0	7.5	1.875	1.875	3.75	1.875	1.875	3.75
V	0	0	7.5	1.875	1.875	3.75	3.75	3.75	0
N	0	0	7.5	3.75	3.75	0	0	0	7.5
D	0	0	7.5	3.75	3.75	0	1.875	1.875	3.75
S	0	0	7.5	3.75	3.75	0	3.75	3.75	0
H	1.875	1.875	3.75	0	0	7.5	0	0	7.5
E	1.875	1.875	3.75	0	0	7.5	1.875	1.875	3.75
P	1.875	1.875	3.75	1.875	1.875	3.75	0	0	7.5
I	1.875	1.875	3.75	3.75	3.75	0	0	0	7.5
K	3.75	3.75	0	0	0	7.5	0	0	7.5
R	3.75	3.75	0	0	0	7.5	1.875	1.875	3.75
A	3.75	3.75	0	0	0	7.5	3.75	3.75	0
L	3.75	3.75	0	1.875	1.875	3.75	0	0	7.5
T	3.75	3.75	0	3.75	3.75	0	0	0	7.5
W	7.5	0	0	7.5	0	0	7.5	0	0
C	7.5	0	0	7.5	0	0	7.5	0	0

[a]Millimolar concentrations of amino acids.

[b]Non-labeled amino acid.

Table S2 Compositions[a] of amino acid mixture solutions for cell-free synthesis of Nrf2 Neh2

Amino acid	for Sample 1			for Sample 2			for Sample 3			for Sample 4		
	NL[b]	^{15}N	$^{13}C/^{15}N$	NL[b]	^{15}N	$^{13}C/^{15}N$	NL[b]	^{15}N	$^{13}C/^{15}N$	NL[b]	^{15}N	$^{13}C/^{15}N$
G	0	0	7.5	0	0	7.5	0	0	7.5	0	0	7.5
F	0	0	7.5	0	0	7.5	1.875	1.875	3.75	1.875	1.875	3.75
P	0	0	7.5	0	0	7.5	7.5	0	0	7.5	0	0
L	0	0	7.5	1.875	1.875	3.75	0	0	7.5	3.75	3.75	0
S	0	0	7.5	1.875	1.875	3.75	1.875	1.875	3.75	0	0	7.5
D	0	0	7.5	1.875	1.875	3.75	3.75	3.75	0	3.75	3.75	0
M	0	0	7.5	3.75	3.75	0	0	0	7.5	0	0	7.5
K	0	0	7.5	3.75	3.75	0	1.875	1.875	3.75	1.875	1.875	3.75
R	0	0	7.5	3.75	3.75	0	3.75	3.75	0	0	0	7.5
A	1.875	1.875	3.75	0	0	7.5	0	0	7.5	3.75	3.75	0
I	1.875	1.875	3.75	0	0	7.5	1.875	1.875	3.75	0	0	7.5
V	1.875	1.875	3.75	1.875	1.875	3.75	0	0	7.5	1.875	1.875	3.75
Y	1.875	1.875	3.75	3.75	3.75	0	0	0	7.5	3.75	3.75	0
Q	3.75	3.75	0	0	0	7.5	0	0	7.5	0	0	7.5
E	3.75	3.75	0	0	0	7.5	1.875	1.875	3.75	3.75	3.75	0
H	3.75	3.75	0	0	0	7.5	3.75	3.75	0	0	0	7.5
T	3.75	3.75	0	1.875	1.875	3.75	0	0	7.5	3.75	3.75	0
W	3.75	3.75	0	3.75	3.75	0	0	0	7.5	0	0	7.5
C	7.5	0	0	7.5	0	0	7.5	0	0	7.5	0	0
N	7.5	0	0	7.5	0	0	7.5	0	0	7.5	0	0

[a]Millimolar concentrations of amino acids.

[b]Non-labeled amino acid.

Table S3. Parameters for measuring and processing the spectra for Ub3A

number of observed complex data points (^{1}H)	1,024
number of observed complex data points (^{15}N)	64
carrier frequency (^{1}H) [MHz]	700.133
carrier frequency (^{15}N) [MHz]	70.952
spectral width (^{1}H) [ppm]	12.57
spectral width (^{15}N) [ppm]	33.01
number of scans	8
recycling delay [seconds]	1 [for ^{15}N-HSQC and HN(CO)],<br>3 (for the others)
apodization function (^{1}H)	squared cosine bell
number of data points after zero filling (^{1}H)	2,048
apodization function (^{15}N)	squared cosine bell
number of data points after zero filling (^{15}N)	128

Table S4. Parameters for measuring and processing the spectra for Nrf2 Neh2

number of observed complex data points (^{1}H)	1,024
number of observed complex data points (^{15}N)	64
carrier frequency (^{1}H) [MHz]	700.233
carrier frequency (^{15}N) [MHz]	70.962
spectral width (^{1}H) [ppm]	11.39
spectral width (^{15}N) [ppm]	20.00
number of scans	32 (for ^{15}N-HSQC),<br>128 (for HN(CO)),<br>16 (for the others)
recycling delay [seconds]	1 [for ^{15}N-HSQC and HN(CO)],<br>3 (for the others)
apodization function (^{1}H)	squared cosine bell
number of data points after zero filling (^{1}H)	2,048
apodization function (^{15}N)	squared cosine bell
number of data points after zero filling (^{15}N)	128

References

Andersson CA, Bro R (2000) The *N*-way toolbox for MATLAB. Chemometrics Intellig Lab Syst 52:1-4

Bro R (1997) PARAFAC. Tutorial and applications. Chemometrics Intellig Lab Syst 38:149-171

Kasai T, Koshiba S, Yokoyama J, Kigawa T (2015) Stable isotope labeling strategy based on coding theory. J Biomol NMR 63:213-221

Korzhnev DM, Ibraghimov IV, Billeter M, Orekhov VY (2001) MUNIN: Application of three-way decomposition to the analysis of heteronuclear NMR relaxation data. J Biomol NMR 21:263-268

Ono S, Kasai T (2018) Efficient constrained tensor factorization by alternating optimization with primal-dual splitting. 2018 IEEE International Conference on Acoustics, Speech and Signal Processing (ICASSP), 15-20 April 2018. 3379-3383

Orekhov VY, Ibraghimov IV, Billeter M (2001) MUNIN: a new approach to multi-dimensional NMR spectra interpretation. J Biomol NMR 20:49-60
